# Pannexin-1 channels, extracellular ATP, and purinergic receptors are essential for CCR5/CXCR4 clustering and HIV entry

**DOI:** 10.1515/nipt-2025-0005

**Published:** 2025-05-23

**Authors:** David Ajasin, Stephani Velasquez, Joy Gibson, Eliana Scemes, Antonio Cibelli, David Spray, Eliseo A. Eugenin

**Affiliations:** Department of Neurobiology, The University of Texas Medical Branch (UTMB), Galveston, TX, USA; Department of Pediatrics Infectious Diseases at Children’s Hospital, Los Angeles, CA, USA; Department of Cell Biology & Anatomy, New York College, Valhalla, NY, USA; Department of Biosciences, Biotechnology, and Environment University of Bari Aldo Moro Bari, Bari, Italy; Dominick P. Purpura Department of Neuroscience and Department of Medicine (Cardiology), Albert Einstein College of Medicine, Bronx, NY, USA; Department of Pathology, Albert Einstein College of Medicine, Bronx, NY, USA

**Keywords:** AIDS, cure, HIV-1, viral reservoirs, hemichannels

## Abstract

**Objective:**

The Human Immunodeficiency Virus-1 (HIV) cell entry has been well characterized with the identification of CD4 as the main receptor and CXCR4 and CCR5 as co-receptors for the virus. However, how the virus uses the cell machinery for entry and infection is still a work-in-progress. Previously, we identified that the Pannexin-1 (Panx-1) channel, extracellular ATP, and purinergic receptors axis are essential for HIV entry and replication in macrophages, but the mechanisms were not fully explored.

**Methods:**

Electrophysiology, ATP quantifications, confocal, HIV entry and replication experiments were used to determine the role of Panx-1 channels in HIV entry.

**Results:**

Here, we identified that HIV or gp120 induces Panx-1 channel opening in association with ATP secretion, purinergic activation, and CCR5/CXCR4/actin clustering to enable HIV entry. Blocking Panx-1 channel opening, ATP secretion, or purinergic signaling prevented co-receptor clustering, HIV entry, and subsequent replication in multiple cell types.

**Conclusion:**

We conclude that gp120 binding to the cell induces Panx-1 opening to promote the clustering of CCR5 or CXCR4 to the site of CD4-gp120 contact to aid viral entry.

## Introduction

Human immuno-deficiency virus-1 (HIV) has become a chronic disease with the introduction of anti-retroviral therapy (ART). However, new infections or viral reactivation remains an important public health issue. An essential stage of the HIV cycle is the entry phase, not only during primary infection but also during the amplification phase of viral reactivation [1]. Critical studies identified the extensive protein-to-protein interactions and networks involved in HIV entry, uncoating, integration, and subsequent replication [2], [3], [4], [5], [6]. However, these reports did not examine the mechanisms of host signaling amplification induced by the virus that does not require protein–protein interactions, such as plasma membrane channels and associated signaling [7].

Overall, the viral protein that mediates HIV entry is the Envelope (Env), which is a highly glycosylated trimer of gp120 (with five conserved domains, C1-C5, and five variable loops, V1-V5), and gp41 heterodimers [8]. Env facilitates viral entry through interactions with multiple host factors, starting with non-specific attachment of virion to the target cell membrane *via* multiple Env interactions with surface factors like heparin sulfate proteoglycan [9] as well as α4β7 integrins [10], 11]. The attachment of the virions to the target cell membrane brings Env close to the CD4 receptor to facilitate HIV entry through the CD4 binding site on gp120, which activates uncharacterized signaling [3]. Afterward, gp120 undergoes conformational changes of the V1/V2 loops that lead to the repositioning of V3 and the formation of the bridging sheet [2]. The conformational changes and formation of the bridging sheets facilitate co-receptor interactions, which induces the activation of gp41 membrane fusion potential. Activation of gp41 induces exposure of its hydrophobic fusion peptide and insertion of the same fusion peptide into the target cell membrane, thereby tethering both the target cell and the virion to each other. gp41, like gp120, is a trimer and, upon insertion into the target cell membrane, folds at the hinge region to bring the amino-terminal (at the target cell membrane) and the carboxyl-terminal (at the viral membrane) of each gp41 subunit together to form a six-helix bundle, which brings both membranes together to form the fusion pore required for the delivery of the viral core into the target cell [12]. However, beyond this detailed information about gp120 binding to CD4 and co-receptors, the question behind the mechanisms of receptor clustering, actin rearrangement, plasma membrane rearrangement, capsid release into the cytoplasm, and intracellular movement remains unexplored.

Previously, our laboratory identified that HIV binding results in the opening of a large plasma membrane channel, Pannexin-1 (Panx-1) channels [13]. Preventing the opening of the channel in response to HIV resulted in low infection [13], but the mechanisms were unknown. Panx-1 are plasma membrane proteins that form large channels that allow ions and various metabolites up to ∼1 kDa to be transported, including ATP, between the intracellular and extracellular space [14], [15], [16], [17], [18], [19]. Panx-1, along with other proteins involved in purinergic signaling, has been shown to be localized at lipid rafts, which are crucial for HIV entry and release [17], 20]. Under normal physiological conditions, Panx-1 channels are closed due to the large conductance of the channels and the potential cell death from prolonged channel opening [21], [22], [23]. However, under some conditions, such as metabolic inhibition, the use of chemotherapeutic medications, and HIV infection, Panx-1 channels are open [13], [24], [25], [26]. In addition, several studies have shown that purinergic receptor activation, which is downstream of Panx-1 opening, is involved in different phases of the HIV replication cycle, especially viral entry [27], 28]. Other pro-HIV pathogenesis events like monocyte maturation, enhancement of monocyte transmigration through the blood-brain barrier (BBB), and pro-inflammation have been associated with elevated extracellular ATP levels and Panx-1 channel opening seen in people living with HIV (PLWH) [7], 18], 27], [29], [30], [31]. Normally, released extracellular ATP acts as a “find me” signal that activates purinergic receptors like P2Y and P2X receptors on the plasma membrane, which regulates the removal of apoptotic cells in healthy conditions but also in inflammation [32], [33], [34]. A compelling study identified both extracellular ATP and UTP in equimolar concentration display both *in vivo* and *in vitro* capacity to recruit phagocytic cells like monocytes, macrophages, and dendritic cells. Also, in that study, the depletion of these extracellular nucleotides or the inhibition of P2Y2 significantly reduced the recruitment of these cells to clear apoptotic cellular debris [32]. Interestingly, some studies have shown that extracellular ATP is also released under healthy conditions, acting as a signaling molecule that enables paracrine cell communication [35]. For instance, a recent study showed that in the brain, astrocytes release neuroactive molecules, including extracellular ATP, to activate purinergic receptors in a calcium-independent manner to regulate synaptic plasticity [36]. However, the contribution of ATP and Panx-1 channels to inflammation and viral infections is still under investigation [35].

In this report, we show that the Panx-1 channel opening induced by the binding of HIV or gp120 is essential for HIV entry and replication by promoting the clustering of HIV receptors on the plasma membrane depending on the tropism of the viral strain. Blocking Panx-1 inhibits the gp120 or HIV-induced co-receptor clustering, reducing HIV entry and subsequent replication.

## Materials and methods

### Primary cells and cell lines

Peripheral blood monocular cells (PBMCs) and monocyte-derived macrophages (MDMs) were isolated from leukopaks obtained from the Gulf Coast regional blood center using Ficoll-paque sequential centrifugation method [37]. Isolated PBMCs were incubated in RPMI media (RPMI, Gibco) supplemented with 10 % heat-inactivated human serum (BIOIVT), 1 % Penicillin-Streptomycin (Pen-Strep, Gibco), 10 mM HEPES (Gibco), IL-2 (Peprotech, 20 U/mL) and activated with PHA (5 μg/mL) for 2–3 days. Post-activation, PBMCs were maintained in PHA-free media. MDMs were differentiated from isolated PBMCs using Dulbecco’s Modified Eagle Medium (DMEM, Gibco) supplemented with 10 % heat-inactivated human serum (BIOIVT), 50 μg/mL Gentamicin (Gibco), 10 μg/mL Ciprofloxacin (Sigma) and rhM-CSF (50 ng/mL, Peprotech) for 7–10 days. All cell lines (HEK 293T, TZM-bL, THP-1, CEM-GFP, U87 transfected with CD4 and CCR5, U87CD4CCR5) were obtained from the NIH HIV reagent program and authenticated at the source. HEK 293T, U87CD4CCR5, and TZM-bL cells were cultured in DMEM (Gibco) supplemented with 10 % FBS (R&D systems), 1 % Penicillin-Streptomycin (Gibco), and 10 mM HEPES (Gibco). THP-1 and CEM-GFP cells were cultured in RPMI (Gibco) supplemented with 10 % FBS (R&D systems), 1 % Pen-Strep (Gibco), and 10 mM HEPES (Gibco). For THP-1 cells, 5 ng/mL Phorbol 12-myristate 13-acetate (PMA) was added to the cells to differentiate them into MDM-like cells for 3 days.

### Viruses, molecular clones, and HIV proteins

Recombinant gp120 proteins (gp120-LAV, gp120-BaL, gp120-JRFL, gp120-IIIB), Plasmids for molecular clones (NL4-3, pNL(AD_8_), and pWT/BaL), and pMM310 plasmid were obtained from NIH HIV reagent, Germantown, MD [38]. The Human Immunodeficiency Virus 1 (HIV-1) NL4-3 AD8 Infectious Molecular Clone (pNL(AD8)), ARP-11346, was contributed by Dr. Freed [38].

### Virus production

For the production of full-length replication-competent viruses, two molecular clone DNAs of either NL4-3 or pNL(AD_8_) were transfected into 10 × 10^6^ HEK 293T cells cultured in Opti-MEM, using Lipofectamine 3,000 (Invitrogen). 6 h post-transfection, Opti-MEM media was replaced with DMEM media (10 % FBS, 1 % Pen-Strep, 10 mM HEPES) and incubated further at 37 °C, 5 % CO_2_, for 24 h. The culture was collected, centrifuged, and filtered through a 0.45 µm filter. The concentration of virus produced was measured *via* p24 ELISA (Perkin Elmer), and aliquots were stored at −80 °C for further use. For pseudo-typed viruses, pWT/BaL molecular clone DNA was co-transfected with pMM310 plasmid expressing β-lactamase enzyme-Vpr (BlaM-Vpr) into 10 × 10^6^ HEK 293T cells using Lipofectamine 3,000. Post-transfection procedure is the same as above.

### HIV infectivity assay

The infectivity of the produced viruses was tested by infecting 2 × 10^6^ TZM-bL cells with 10 ng/mL of NL4-3, pNL(AD_8_), and pseudo-typed viruses and incubated for 36 h. Cells were collected and lysed, and luciferase activity was determined according to the Promega luciferase assay kit protocol using a plate reader (PerkinElmer EnVision system).

### HIV infection and replication

5 × 10^6^ PHA-activated PBMCs were plated in each well in 6 well-plates and designated wells were inoculated with 10 ng/mL of NL4-3 for 15 days, and 500 µL media was collected and replenished (with fresh media) every 3 days till day 15 post-infection. 2 × 10^6^ MDMs were plated in each well of 6 well plates and inoculated with 10 ng/mL of pNL(AD_8_) for 28 days, and media was collected and replenished (with fresh media) on days 3, 7, 11, 15,19, 23, and 28 post-infection. 5 × 10^6^ CEM-GFP cells were infected with NL4-3 for 15 days. Media collection and replenishment (with designated conditions) were done every 3 days post-infection until day 15. We have to emphasize that all the above cells were subjected to the following pre-treatment conditions: media only (control), 500 µM Probenecid (Sigma), 300 µM ^10^Panx peptide, or 300 µM scrambled peptide for 30 min before HIV infection (see Table 1 for peptide sequence) synthesized by Genscript. Virus replication was monitored with HIV-p24 ELISA.

**Table 1:** Sequence of peptides used in this study.

Peptide name	Peptide sequence	Manufacturer
10-Panx peptide	WKQAAFVDSY	Genscript
Scrambled peptide	FSVYWAQADK	Genscript

### HIV-p24 ELISA

To determine p24 levels from either virus production from HEK 293T cells or virus replication from primary or CEM-GFP cells, collected media were centrifuged at 2000×*g* for 10 min at 4 °C, and the supernatants were collected and filtered through 0.45 µm filter. Using appropriate dilutions, supernatants were diluted in DMEM media to set dilutions and quantified using HIV-1 p24 ELISA (XpressBio or Perkin Elmer) to generate a standard curve and determine viral concentrations.

### ATP measurement

Extracellular ATP levels in supernatants from PBMCs, MDMs, and CEM-GFP cells were determined. Supernatants were collected from multi-day replication experiments from 5 × 10^6^ PHA-activated PBMCs (every 3 days post-infection), 2 × 10^6^ MDMs (at days 3, 7, 11, 15, 19, 23 and 28 post-infection) and 5 × 10^6^ CEM-GFP cells (every 3 days post-infection). Briefly, cells were pre-treated with media only (control), 500 µM Probenecid (Sigma), 300 µM ^10^Panx peptide, or 300 µM scrambled peptide for 30 min and inoculated with 10 ng/mL NL4-3 (for PBMCs and CEM-GFP) and pNL(AD_8_) for MDMs, and virus replication was allowed to go on for 15 days (for PBMCs and CEM-GFP cells) and 28 days (for MDMs). Collected supernatants (200 µL) were centrifuged and filtered through a 0.45 µm filter, and appropriate dilutions in media were done before subjecting the samples to ATP quantification assay, ATP luciferase/luciferin-based enzyme assay (ATPlite, PerkinElmer). The assay was done according to the manufacturer’s protocol.

### GFP reading

CEM-GFP cells from multi-day replication experiments were centrifuged at 300×*g*, 4 °C for 10 min. Collected cells were resuspended in PBS (supplemented with 2 % FBS and 1 mM EDTA), and 5 × 10^4^ cells were added to designated wells of 96 well plates. Absorbance readings (excitation at 395 nm wavelength and emission at 508 nm wavelength) were collected using a plate reader (EnVision, PerkinElmer) for each condition (control, HIV, HIV+500 µM Probenecid, HIV+300 µM ^10^Panx peptide, and HIV+300 µM Scr peptide).

### Etd uptake assay

The opening of Panx-1 channels was determined *via* the Etd dye uptake assay. Briefly, 1 × 10^6^ PHA-activated PBMCs were plated per well and designated wells pre-treated with 500 µM Probenecid, ^10^Panx1 peptide (300 µM), or Scr peptide (300 µM) for 30 min. gp120 (200 ng/ml) or HIV (10 ng/mL NL4-3) was added to designated wells and incubated at 37 °C for 30 min. 2 × 10^5^ cells for each condition were collected and plated in fresh 24 well plates and incubated with 1/10,000 dilution of Hoescht for 30 min. EtBr (5 µM) was added to each well and positioned in the live cell imaging Zeiss epi-fluorescent microscope to collect images at the following time points: 5, 10, 15, 20, and 30 min. The mean fluorescent intensity (MFI) of each channel (Hoechst and Etd) per image was determined, and the percentage of Etd uptake of cells was determined (as Etd MFI/Hoechst MFI × 100).

### CCR5/CXCR4 plasma membrane distribution

PBMCs were isolated and divided into two halves, with one half for PBMC (1 × 10^6^ cells/well) and the rest differentiated to MDMs (2 × 10^5^ cells/well) as described. Designated wells were pre-treated with media-only (control), 500 µM Probenecid, ^10^Panx-1 peptide (300 µM), or Scr peptide (300 µM) and incubated at 37 °C, 5 % CO_2_ for 30 min. The following were added to designated wells: gp120 (200 ng/mL) or HIV, 50 ng NL4-3 (for PBMCS), or pNL(AD_8_) (for macrophages)and incubated at 37 °C for 5-, 10-, 15-, 20-, and 30-min. Cells were fixed with 4 % paraformaldehyde (PFA) for 15 min at room temperature and PBMCs were centrifuged. Cells were washed with 1X PBS, permeabilized with 0.1 % Triton-X in 1X PBS for 3 min at room temperature, and incubated with 1X SBS blocking buffer (50 mM EDTA, 1 % Fish gelatin, 0.1 g BSA, 1 % Horse serum, 5 % Human serum) for at least 1 h at room temperature. Cells were incubated with primary antibodies (2.5 μg/mL of anti-CXCR4, anti-CCR5-FITC, and anti-Panx-1) at 4 °C overnight and washed with 1X PBS. Slides were incubated with corresponding secondary antibodies (5 μg/mL) at room temperature for at least 1 h. As a control, isotyped primary antibodies and corresponding secondary antibodies were used. Cells were washed with 1X PBS and mounted with Prolong Gold with DAPI (Invitrogen). Images were collected with a Nikon A1 confocal microscope. Co-receptors and Panx-1 CCR5/CXCR4/Panx-1 surface distribution or clustering, as well as colocalization, were determined and analyzed with the Nikon Elements AR analysis software. Co-receptor clustering was determined as a function of aggregation of CCR5/CXCR4/Panx-1 compared to control conditions, where all three are in diffuse conditions on the plasma membrane. For colocalization, Pearson coefficient value ≥0.5 represents colocalization, and <0.5 represents no colocalization.

### HIV entry assay

HIV entry was determined in two ways. The first entry assay used a CCF4-AM kit (K1085, Invitrogen), and the second used TZM-BL cells to perform a single-cycle infection assay. For the CCF4-AM entry assay experiments, MDMs or PMA differentiated THP-1 cell lines were plated in 12 well plates and pre-treated with 500 µM Probenecid, ^10^Panx-1 peptide (300 µM), or Scr peptide (300 µM) and incubated at 37 °C, 5 % CO_2_ for 30 min. Cells were infected with 200 ng/mL pseudo-typed virions (produced from co-transfection of two plasmids: pWT/BaL and pMM310). Cells were centrifuged to perform spinoculation at 2,900 rpm, 37 °C, for 2 h and incubated for 4, 8 or 12 h at 37 °C, 5 % CO_2_ (12 h only for THP-1). Media was replaced with FluoroBrite DMEM media (supplemented with 10 % human serum for MDMs or fetal bovine serum for THP-1, 50 μg/mL Gentamicin, 10 μg/mL Ciprofloxacin, and 10 mM HEPES) plus CCF4-AM substrate. Cells were incubated overnight at 12 °C to allow for CCF4 cleavage by the BlaM enzyme. Using the EnVision PerkinElmer plate reader, fluorescence was measured with excitation at 410 nm and emission at 460 and 528 nm for the blue and green signals from the cells. The percent of HIV entry was determined by blue absorbance/green absorbance × 100. For the single-cycle infection assay, TZM-BL cells were plated and pre-treated as before. Cells were infected with 200 ng/mL of pNL(AD8) virus and incubated at 37 °C for 36 h. Cells were collected, lysed, and examined for luciferase activities using a Promega luciferase assay kit.

### Early and late reverse transcriptase qPCR assay

To evaluate the immediate post-entry phase of the HIV replication cycle, reverse transcriptase (RT) products, both early and late, were examined and quantified as a read-out of successful entry. MDMs were treated as described in the result section with 100 ng/mL of full virus pNL(AD8) for 48 h and 72 h [39]. Cells were collected, washed, and total DNA was extracted using the DNeasy Blood and Tissue Kit (QIAGEN). Extracted DNA was normalized, and 500 ng DNA/50 µL qPCR prep was used to perform qPCR to detect both Early and Late RT products using 300 nM primers (see Table 2 for primers sequence), SyBr-Green assay kit (Thermo Scientific) and AB StepOnePlus Real-time PCR machine. Cycling conditions: 2 min at 50 °C, 10 min at 95 °C, 40 cycles of 15 s at 95 °C, and 1 min at 60 °C [39].

**Table 2:** Sequence of primers used in this study.

Primer name	Primer sequence	Manufacturer
Early RT forward	GTGCCCGTCTGTTGTGTGAC	IDT DNA
Early RT reverse	GGCGCCACTGCTAGAGATTT	IDT DNA
Late RT forward	TGTGTGCCCGTCTGTTGTGT	IDT DNA
Late RT reverse	GAGTCCTGCGTCGAGAGAGC	IDT DNA

### Electrophysiology

Cells were plated on glass coverslips 1–2 days prior to recordings. Whole-cell patch-clamp recordings were performed on cells bathed in HEPES-supplemented phosphate-buffered saline (H-PBS) containing (mM): NaCl 147, HEPES 10, CaCl_2_ 2, MgCl_2_ 1, and KCl 2.7, 8 mM Na_2_HPO_4_, and 2 mM KH_2_PO4 pH 7.4. The pipette solution contained (mM): CsCl 130, EGTA 10, HEPES 10, CaCl_2_ 0.5, pH 7.4. Recording of the activation of Panx1 channels by voltage was performed by applying 1 s −10 mV pulse, followed by 12-s voltage ramps from a holding potential of −70 mV to +70 mV, followed by 1-s steps to 0 and −30 mV to evaluate possible tail currents. To analyze the participation of Panx-1 channels, U87 expressing CD4/CCR5 cells were exposed to either control (C) or different concentrations of gp120 were used depending on the threshold (gp120 containing solution, 56 °C heat inactivated gp120 (10 ng/mL)+1 µM ATP or 1 μg/mL gp120 protein+1 µM ATP, respectively) for 1–2 min and then rinsed with H-PBS. Electrophysiological recordings were accomplished using an Axopatch 1-C amplifier, and pClamp10 software was used for data acquisition and analysis. Changes in peak conductance induced by the proteins were normalized to those recorded in H-PBS alone prior to exposure to the agents and expressed as fold changes.

### Statistical analysis

Statistical analyses were conducted using GraphPad Prism Version 10.4.0 (GraphPad Software, San Diego, CA). Data are presented as Mean±SD or SEM (where indicated). The statistical significance was assessed using the ANOVA test, unpaired nonparametric two-tailed Student’s *t*-test, or the area under the curve, with a p≤0.05 to be considered statistically significant.

### Data availability

Data are available upon reasonable request.

## Results

### gp120 induces the opening of Panx-1 channels

Despite our knowledge about HIV cell binding, entry, and replication, few studies have addressed the contribution of other host proteins/lipids beyond the main receptor and co-receptors, including receptor/co-receptors aggregation, lipid raft accumulation, and actin rearrangement. However, how these other host components help viral entry and other downstream events critical to productive HIV infection or reactivation is unknown.

**Figure 1:**
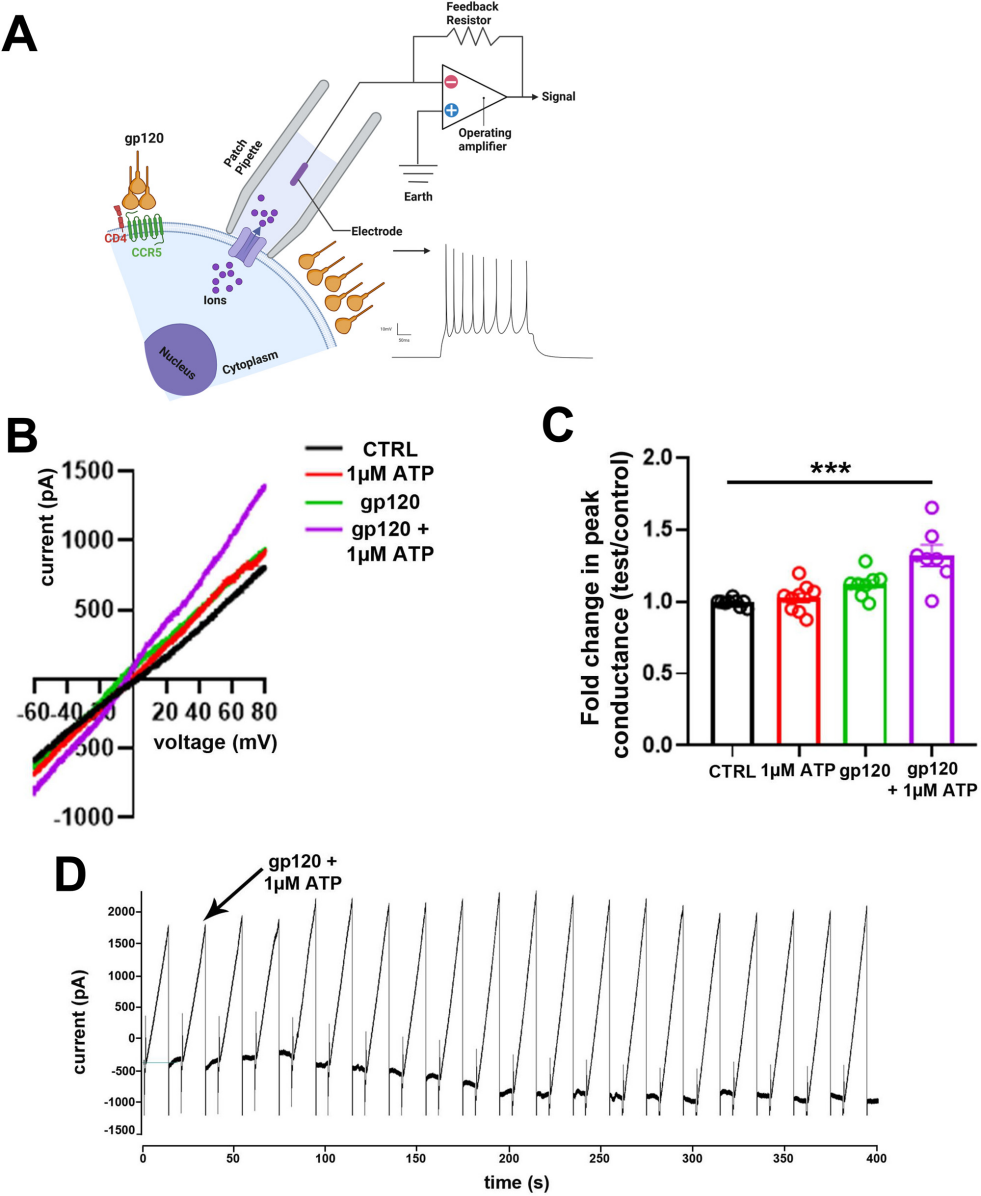
gp120 protein induces opening of Panx-1 channels, (A) schematic of electrophysiological recording from U87-CD4-CCR5 cells. (B) Current/voltage (I/V) curves from U87CD4CCR5 cell patch clamp experiments in control (CTRL), ATP, gp120 only, and gp120+ATP, n=3. (C) Quantification of fold change relative to peak conductance (***p≤0.005 compared to control conditions, n=8). (D) Representative recording of patch-clamped U87CD4CCR5 cells treated with gp120+1 µM ATP over 4,000 s. Statistics: nonparametric *t*-test, n=8, ***p value ≤0.001.

The interaction of gp120 with CD4 and CCR5 or CXCR4 evoked plasma membrane currents that have distinct signaling compared to chemokine binding to the sample receptors, including calcium-activated potassium channels, chloride, and calcium permeant non-selective cation channels [40]. Our data in macrophages and PBMCs identified that some of these channels are Panx-1 channels [7], but their electrophysiological properties are unknown. Thus, we performed whole-cell path-clamp recording using the human astrocytoma cell line transfected with CD4 and CCR5 (see Figure 1A, cartoon) in response to gp120 application (10 ng/mL). We measured changes in peak conductance induced by voltage ramps before and after adding gp120 (10 ng/mL), ATP (1 µM), or both, normalized to PBS-alone.

Control conditions (PBS), 1 µM ATP, or gp120 (10 ng/mL) application alone did not change the I/V curves upon changes in voltage ramps (Figure 1B). However, the combination of gp120 and ATP resulted in a significant increase in currents compared to individual treatments (Figure 1B, gp120+ATP). The determination of the peak of conductance confirmed the data that gp120 and ATP together increase Panx-1 channel opening (Figure 1C and D, n=8 independent experiments). The change in current amplitude of control 1.0±0.02 folds, 1 µM ATP 1.0±0.2 folds, and gp120 1.18±0.17 folds (JRFL, IIIB, or 10 ng/mL or 1 μg/mL). The combination of gp120 and ATP resulted in channel opening (Figure 1B and C, representative recordings in Figure 1D). However, the combined treatment of gp120 (1 μg/mL) plus ATP (1 µM) resulted in increased peak conductance of 1.35±0.3 folds (Figure 1C, n=8, ***p≤0.005 compared to control). Overall, the combination of gp120 and ATP synergizes the Panx-1 opening.

### Recombinant gp120 or HIV induces Panx-1 channel opening

To address the time course of Panx-1 channel opening, primary cultures of human PBMC were subjected to an Ethidium uptake assay (Etd, 5 µM). The Etd dye only crosses the plasma membrane in healthy cells by the opening of large ionic channels such as Connexin hemichannels or Panx-1 channels [13], 24]. For all the subsequent experiments, no changes in Panx-1 expression were detected in PBMCs, macrophages, or CEM-GFP cells upon HIV or 200 ng/mL gp120-LAV exposure (Supplementary Figure 1, representative data for 10 ng/mL NL4-3 for PBMCs and CEM-GFP, and pNL(AD_8_) for MDM).

**Figure 2:**
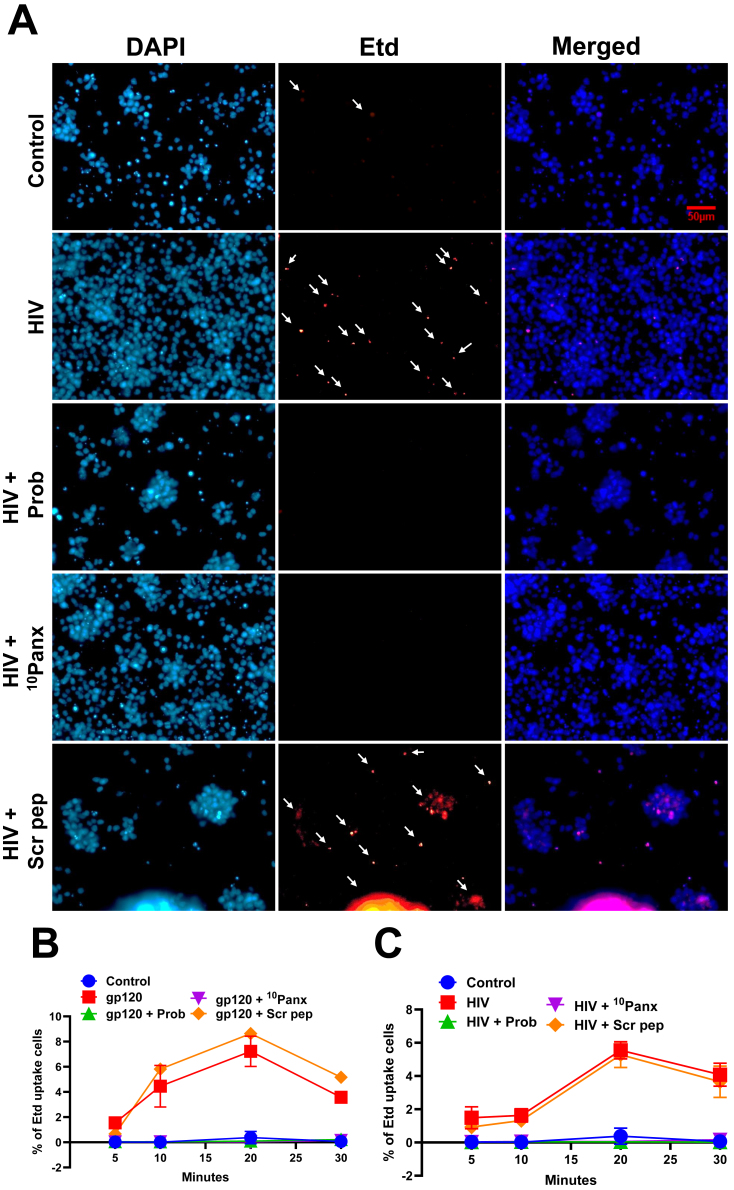
Exposure of PBMCs to gp120 or HIV induces Panx-1 channel opening. (A) Representative image of PHA-activated PBMCs plated and subjected to the following pre-treatment for 30 min (PBS-only for control and gp120/HIV only), 500 µM Probenecid, 300 µM Panx-1 mimetic peptide, ^10^Panx peptide, and 300 µM scrambled peptide (Scr pep). Afterwards, cells were exposed to gp120 (200 ng/mL) or NL4-3 (10 ng/mL). Etd was added, and Etd uptake was monitored with live cell imaging. Image is at 10 min post-addition of Etd, n=3. White arrows indicate cells with Etd uptake. (B) Quantification of the percentage of Etd uptake in all gp120-treated PBMCs relative to DAPI. (C) Quantification of percentage of Etd uptake in all HIV infected PBMCs relative to DAPI displayed as % of Etd uptake cells is determined by number of cells positive for Etd/total number of cells (DAPI positive) × 100. Statistics: area under the curve p value ≤0.05 from 10 to 30 min; control versus. gp120/HIV, control versus. gp120/HIV+Scr pep, gp120/HIV versus. gp120/HIV+Prob/^10^Panx. Data are represented as mean±SEM, n=3.

Human PBMCs in untreated conditions showed low to undetectable Etd uptake, indicating that the plasma membrane is impermeable to the dye (Figure 2A, Control). Conversely, gp120 or HIV exposure induced Etd uptake (Figure 2A, for HIV, and gp120 and HIV shown in Supplementary Figures 2 and 3, respectively). Panx-1 channel opening induced by gp120 or HIV was sensitive to the pre-application of Probenecid (Figure 2A, Prob, 500 µM) and the mimetic peptide to the extracellular portion of Panx-1 (Figure 2A, ^10^Panx-1, 300 µM), but not to the scrambled peptide (Figure 2A, Scr pep, 300 µM). Quantification of Etd uptake experiments indicated that gp120 (Figure 2B, all points show significant Etd uptake from 10 min to 25 min, n=4, p≤0.005 compared to control conditions) or HIV (Figure 2C, all points show significant Eth uptake from 10 min to 30 min, n=4, p≤0.005 compared to control conditions) triggers the opening of Panx-1 channels. The time course of Panx-1 channel opening induced by gp120 or HIV was significant after 10 min and remained high compared to control up to ∼30 min of recording, the last point assayed (Figure 2B and C, respectively). No cell death or loss was observed in all cultures analyzed by trypan blue or TUNEL staining (data not shown). These results indicate that gp120 or HIV induces a long-term opening of Panx-1 channels.

### Panx-1 channel opening induced by HIV or gp120 results in ATP release

**Figure 3:**
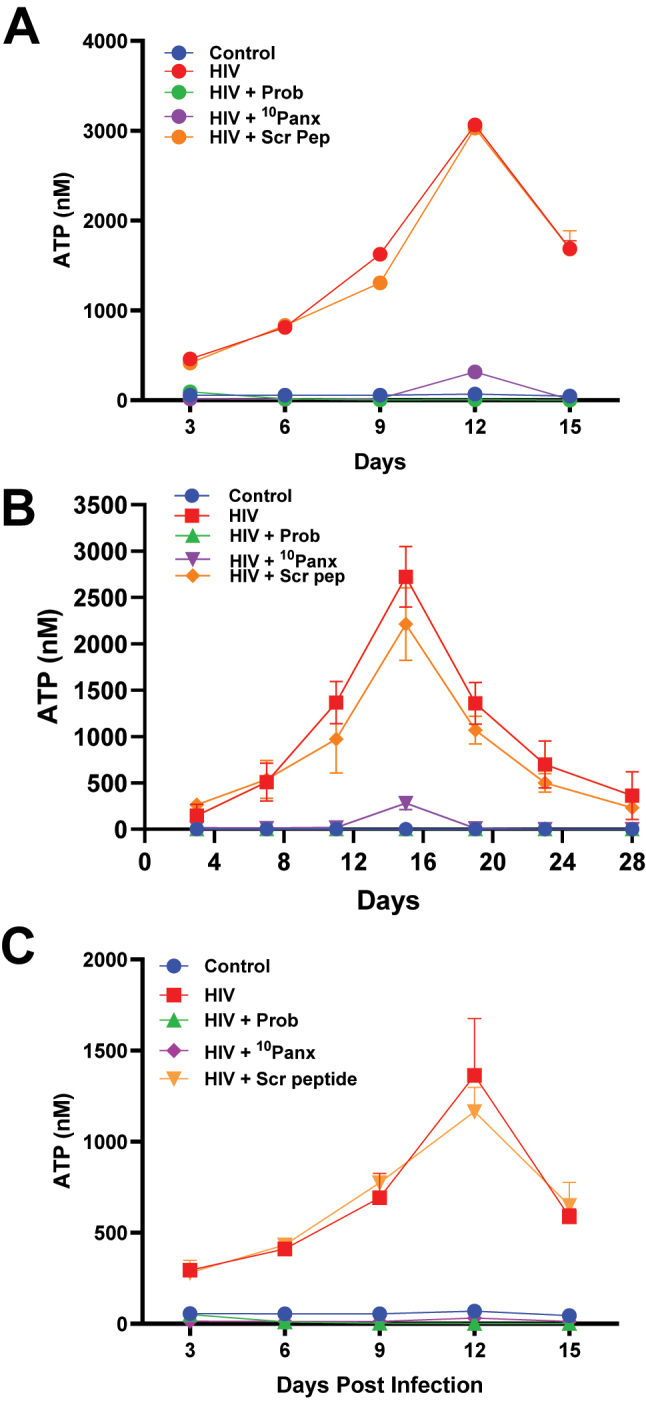
Blocking the Panx-1 channel inhibits ATP release in PBMCs, MDMs, and CEM-GFP cells. (A) PHA-activated PBMCs were subjected to the following pre-treatments for 30 min PBS-only (for control and HIV only), 500 µM Probenecid, 300 µM Panx-1 mimetic peptide, ^10^Panx peptide, and 300 µM scrambled peptide (Scr pep). Followed by replication of HIV after inoculation with NL4-3 (10 ng/mL), and media collected every 3 days until day 15 post-infection and ATP levels determined with ATP luciferase/luciferin-based enzyme assay, n=3. (B) MDMs were subjected to the following pre-treatments for 30 min PBS-only (for control and HIV only), 500 µM Probenecid, 300 µM Panx-1 mimetic peptide, _10_-Panx peptide, and 300 µM scrambled peptide (Scr pep). Followed by replication of HIV after inoculation with pNL(AD_8_) (10 ng/mL) and media collected on days 3, 7, 11, 15, 19, 23, and 28 post-infection, followed by ATP luciferase/luciferin-based enzyme assay to determine ATP levels, n=3. (C) CEM-GFP cells were subjected to the following pre-treatments for 30 min media-only (for control and HIV only), 500 µM Probenecid (Prob), 300 µM Panx-1 mimetic peptide, (^10^Panx), and 300 µM scrambled peptide (Scr pep). Followed by replication of HIV after inoculation with NL4-3 (10 ng/mL) for 15 days, and media collected every 3 days until day 15 post-infection. Extracellular ATP levels in media were determined with ATP quantification assay using luciferase/luciferin-based enzyme assay, n=3. Statistics: area under the curve show significant difference p value <0.05 control versus. HIV, and control versus. HIV+Scr pep, also, there is significant difference, p value <0.05 HIV versus. HIV+Prob and HIV versus. HIV+ ^10^Panx-1. Data are represented as mean±SD.

A critical finding in human macrophages is the ATP release into the extracellular space in response to HIV or SARS-CoV-2 infection by a mechanism solely dependent on Panx-1 channel opening [7], 13], 15]. Further, the gp120-induced opening of Panx-1 channel requires the presence of extracellular ATP, as determined in Figure 1, which indicates the essential interplay between Panx-1 channel opening and extracellular ATP. To examine the time course of ATP secretion, we quantified the extracellular concentration of ATP in a multi-day HIV replication experiment using human PBMCs (Figure 3A), macrophages (Figure 3B, MDM), and CEM-GFP cells (Figure 3C, human T4-lymphoblastoid cell line).

CEM-GFP, PBMCs, and MDM cultures were treated with media-only or HIV (10 ng/mL NL4-3 for PBMCs/CEM-GFP, and pNL(AD_8_) for MDM), and ATP concentrations in the media were determined at different time points (Figure 3A for PBMC, 3B for macrophages, and Figure 3C for CEM-GFP). The time course of ATP release for all three cell types in response to HIV was similar, with significant extracellular ATP levels with a peak of secretion around 12 days post-infection (Figure 3A–C all points are significant from 6–7 to 21 days, n=3–5, p≤0.005 compared to control conditions). Low to no ATP secretion was detected in the control conditions (Figure 3A–C). ATP release induced for the virus was unaffected by the scrambled peptide (Figure 3A–C, Control or Scr pep). In contrast, the pre-incubation with Panx-1 channel blockers such as Probenecid (Prob, 30 min) or the mimetic Panx-1 peptide (^10^Panx-1, 30 min) before the HIV inoculation prevented ATP release into the media (Figure 3A–C). In the case of CEM-GFP cells, we observed similar results when compared to PBMCs and MDM, however, with lower overall ATP concentration. ATP secretion was similarly sensitive to Panx-1 blockers (Figure 3C Probenecid, Prob; mimetic Panx-1 peptide, ^10^Panx-1).

### Panx-1 channel opening induced by HIV is essential for HIV entry

**Figure 4:**
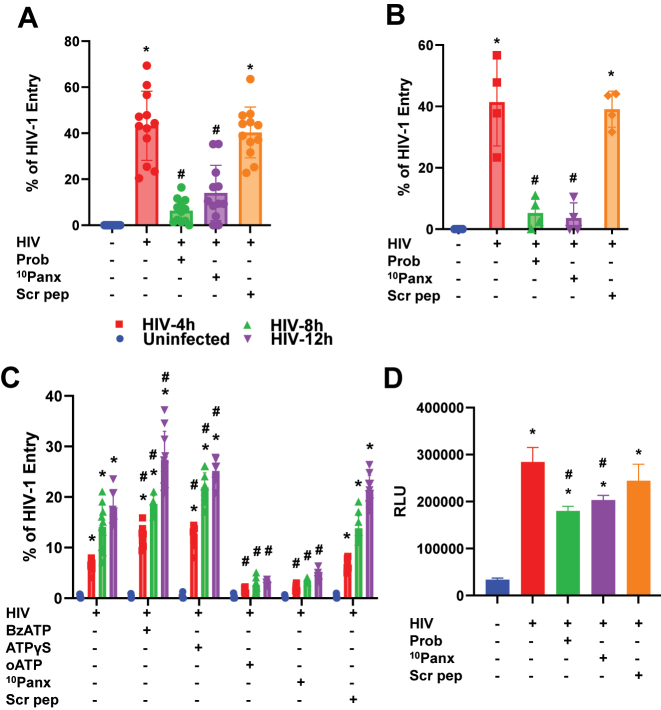
Pannexin-1 and released extracellular ATP contribute to HIV entry in MDMs and cell lines. (A) Pre-treated MDMs were infected with β-lactamase containing pseudo-typed virus (pWT/BaL-pMM310) for HIV entry assay, n=12. (B) Pre-treated THP-1 cells were infected with β-lactamase containing pseudo-typed virus (pWT/BaL-pMM310) for HIV entry assay, n=4. (C) MDMs from an individual was pre-treated with BzATP (300 µM), ATPγS (15 µM), oATP (200 µM), _10_-Panx peptide (300 µM), and scrambled (Scr) peptide (300 µM), and inoculated with β-lactamase containing pseudo-typed virus (pWT/BaL-pMM310, 200 ng/mL) to test for HIV entry at 4-, 8-, and 12-h post-inoculation, n=4. % of HIV-1 Entry is determined as; number of cells positive at 460 nm/number of cells positive at 528 nm × 100. (D) Relative Light Unit (RLU) of pre-treated TZMbL cells inoculated with 10 ng/mL pNL(AD_8_), to HIV entry on viral transcription, n=3. Statistics: nonparametric *t*-test for figures A, B, D, and Two-Way ANOVA for figure C. Data are represented as mean±SD, *p≤0.005 compared to controls and #p≤0.005 compared to HIV.

Two groups have shown that both purinergic receptors and Panx-1 channel opening participate in HIV entry through coordinated ATP secretion and purinergic signaling [13], 27], 31]. However, the mechanisms are not fully understood. To determine if Panx-1 opening is needed for HIV entry, macrophages (Figure 4A) and THP-1 (Figure 4B) cultures were pre-treated with 500 µM Probenecid, 300 µM ^10^Panx, or 300 µM Scr pep for 30 min and then inoculated with 200 ng/mL of β-lactamase containing viral particles (pWT/BaL-pMM310 pseudo-typed virus for 4 h) to determine entry. The percentage of viral entry was determined by assessing the number of total cells (green) relative to the blue cells (HIV entry events) [13].

Human macrophages exposed to PBS (control) did not show any entry signal as expected (Figure 4A, blue points, n=12). However, macrophage cultures inoculated with pseudo-typed HIV (pBaL/WT-pMM310 virus) led to 43.21±4.33 % entry (Figure 4A, n=12, red points, HIV, *p≤0.005 compared to control conditions). Pre-incubation of the cultures with Probenecid (Prob) or mimetic Panx-1 peptide (^10^Panx-1) prevented HIV entry (Figure 4A, n=12 respectively, #p≤0.005 compared to the HIV condition). The scrambled peptide (Scr pep) was comparable to the HIV condition (Figure 4A, n=12, *p≤0.005 compared to control conditions, fifth column, Scr pep). Due to the variability of HIV entry in primary cells, experiments in THP-1 cells (a human leukemia monocytic cell line, ATTC-TIB-202) treated with PMA (5 ng/mL) to generate macrophage cell-like cultures were performed (Figure 4B). No cleavage of the CCF4-AM dye in response to infection was observed in uninfected cultures as expected (Figure 4B, blue dots). HIV entry was detected in HIV-inoculated conditions (Figure 4B, HIV, n=4, *p≤0.005 compared to control conditions). Pre-incubation of the cultures for 30 min with Prob or ^10^Panx peptide significantly prevented HIV entry (Figure 4B, HIV+Prob or HIV+ ^10^Panx, respectively, n=4, *p≤0.005 HIV compared to control, and #p≤0.005 compared to HIV conditions), while the scrambled peptide did not affect HIV entry (Figure 4B, HIV+Scr pep, n=4, *p≤0.005 HIV+Scr pep compared to control).

To demonstrate the role of Panx-1 channels and purinergic signaling in HIV entry, we examined whether activation or blocking of purinergic receptors altered HIV entry (Figure 4C). Briefly, HIV entry was evaluated after 4, 8, and 12 h post-infection with the pseudo-typed HIV in primary macrophage cultures (Figure 4C). No entry was detected in uninfected cultures (Figure 4C), while HIV exposure resulted in entry at all time point examined (Figure 4C, HIV, 6.26±0.3. red bars). To further activate purinergic receptors, before infection, we pre-treated for 5 min with BzATP (Benzoylbenzoyl-ATP, 2′(3′)-O-(4-Benzoylbenzoyl) adenosine 5′-triphosphate, 300 µM), a P2X_7_ non-hydrolyzable agonist, which resulted in higher HIV entry, 11.9±0.9, compared to HIV alone at all the time points examined (Figure 4C, BzATP, n=4, *p≤0.005 compared to control conditions, and #p≤0.005 compared to HIV condition). However, we must note that ∼40 % of the experiments (n=5) treated with BzATP resulted in massive apoptosis, probably due to the heterogeneous expression of P2X7 and other purinergic receptors in the human population (data not shown). In addition, the pre-incubation with ATPγS (15 µM), a non-hydrolyzable analog of ATP that acts as a P2 agonist, before exposure to HIV, also resulted in higher HIV entry, 12.04±0.84, compared to HIV alone at all time points (Figure 4C, ATPγS, n=4, *p≤0.005 compared to control conditions, and #p≤0.005 compared to HIV condition). In contrast, the pre-treatment with purinergic blockers, such as oxidized ATP (oATP), prevented HIV entry (Figure 4C, oATP, n=4, #p≤0.005 compared to HIV condition) with comparable magnitude to when we blocked Panx-1 channel opening with the mimetic ^10^Panx peptide (Figure 4C, ^10^Panx, n=4, #p≤0.005 compared to HIV condition). No changes in HIV entry were observed with the Scrambled peptide (Figure 4C, Scr pep).

Further, to examine how Panx-1 mediated entry affects another phase of the viral life cycle, we evaluated HIV transcription in a reported cell line, TZM-bL, which expresses Luciferase upon HIV successful entry and transcription [41]. In the uninfected condition, a minimal Luciferase signal was detected (Figure 4D. 33,927±1,097). HIV infection for 36 h significantly enhanced Luciferase signal over control (284,133±12,797, n=4, *p≤0.005 compared to control conditions). The pre-addition of Probenecid (Prob) or the mimetic Panx-1 peptide (^10^Panx) prevented HIV-driven Luciferase expression, 180,073±2,073 and 203,200±2,503, respectively, which indicates a significant block of HIV entry (Figure 4D, n=4, *p≤0.005 compared to control conditions, and #p≤0.005 compared to HIV condition). Scrambled peptide did not affect HIV-induced Luciferase expression induced by the virus (Figure 4D, Scr pep, *p≤0.005 compared to control conditions). Overall, our data indicates that the axis Panx-1/ATP/purinergic receptor is essential to regulate HIV entry as well as replication.

### Opening of Panx-1 channels induced by gp120 or HIV triggers CXCR4 and CCR5 clustering

A critical question is how Panx-1 opening, ATP secretion, and purinergic activation enhanced HIV entry and subsequent replication. Several studies have shown that HIV entry requires clustering of the co-receptor(s) to specific regions of the plasma membrane [42], [43], [44], [45], [46], [47]; however, the mechanism behind this signaling is unknown.

**Figure 5:**
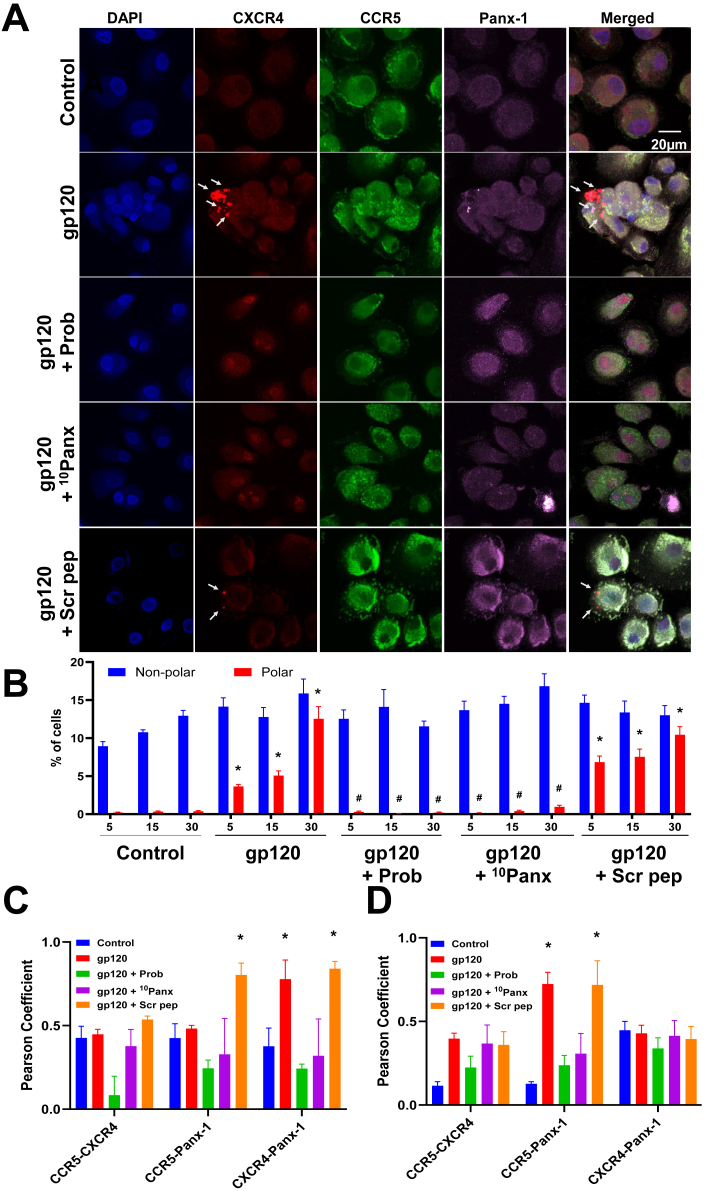
gp120 induce CXCR4 clustering in PBMCs through Panx-1 opening. (A) Confocal images of PHA-activated and pre-treated PBMCs exposed to LAV gp120 (200 ng/mL) and stained for DAPI, CXCR4, CCR5, and Panx-1 proteins to visualize co-receptor distribution in all conditions examined. Arrows indicate co-receptor clustering, n=3, Scale bar=20 µm. (B) Quantification of cells with CXCR4 clustering in response to examined conditions from confocal imaging in (A). Statistics: Two-Way ANOVA, data are represented as mean±SD, *p≤0.005 compared to untreated and #p≤0.005 compared to gp120 only condition. (C) Pearson colocalization coefficient of gp120-LAV treated cells in (A) above shows Panx-1 colocalizing (>0.5) in gp120 only and gp120+Scr peptide conditions and no colocalization in Probenecid and ^10^Panx peptide conditions. (D) Pearson colocalization coefficient of BaL gp120 treated cells in Supplementary Figure 5 shows Panx-1 colocalizing (>0.5) in gp120 only and gp120+Scr peptide conditions and no colocalization in Probenecid and ^10^Panx peptide conditions.

**Figure 6:**
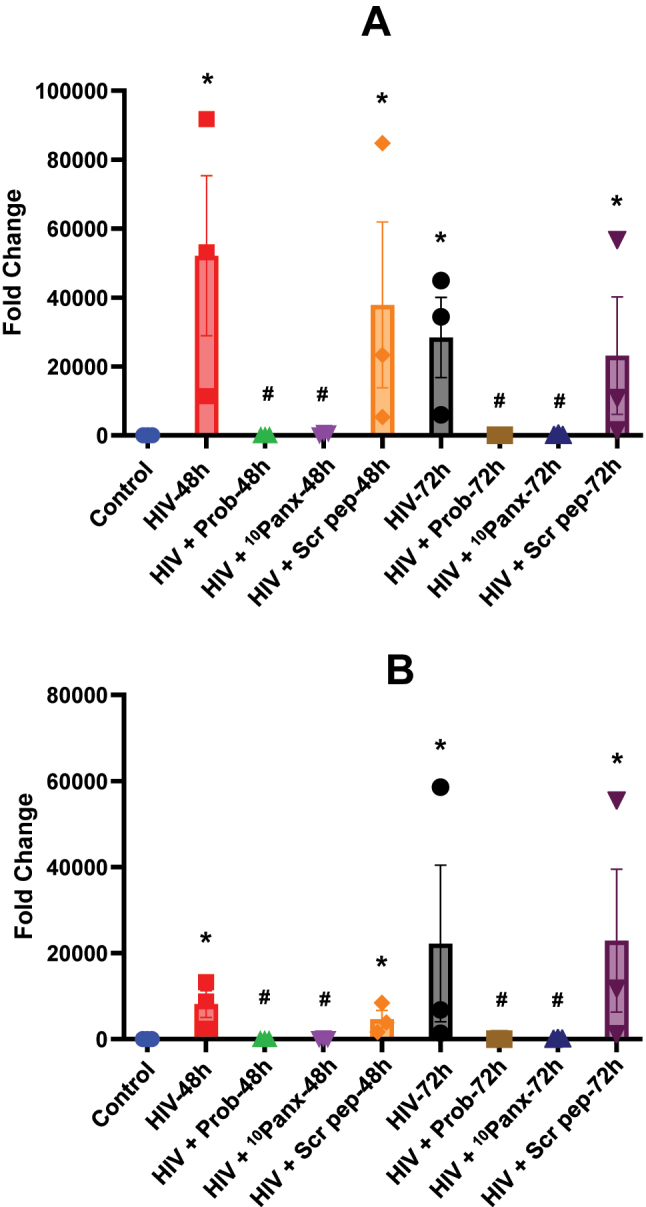
Reverse transcriptase (RT) product synthesis is reduced by blocking of the Panx-1 channel opening. Fold change determined *via* qPCR ascertains the level of early (A) or late (B) RT products synthesized from pre-treated and HIV-infected macrophages at 48- and 72-h post-infection, n=3. Data are represented as mean±SD, *p≤0.005 compared to control.

To examine whether the Panx-1/ATP/purinergic axis plays any role in chemokine receptors (CCR5 and CXCR4) clustering induced by gp120 or HIV, we used confocal microscopy to quantify the density and distribution of these receptors at the plasma membrane. PBMCs and macrophage cultures were exposed to X4 or R5 gp120 (LAV or BaL, 200 ng/mL) or viruses (NL4-3 or pNL(AD_8_), 50 ng/mL, respectively) for 5–30 min to examine early stages of chemokine receptor distribution and HIV entry (Figure 5 and Supplementary Figures 5–7). In control conditions, there was little to no CXCR4, CCR5, or Panx-1 clustering observed (Figure 5A, control, and 5B, quantification) in both PBMCs and MDMs. gp120-LAV treatment of PBMCs induced CXCR4 clustering (Figure 6A, see arrows, gp120, and 5B, quantification, *p≤0.005 compared to control conditions). Experiments using macrophage cultures showed similar clustering induced by BaL-gp120 (Supplementary Figure 5A, gp120, and 5B, quantification, *p≤0.005 compared to control conditions). Pearson colocalization index indicates that gp120-LAV and BaL, induced higher colocalization of the co-receptors with Panx-1 (CXCR4/Panx-1 and CCR5/Panx-1 respectively) that was prevented by Probenecid (Prob) or the ^10^Panx peptide (^10^Panx) (Figure 5C and D, respectively, *p≤0.005 compared to control conditions, n=4). In contrast, the scrambled peptide (Scr pep) did not prevent the gp120-induced CXCR4/CCR5/Panx-1 clustering on the plasma membrane (Figure 5A, C and D and Supplementary Figure 5A and B, gp120+Scr pep). In conclusion, gp120 induces Panx-1 opening, ATP secretion, and purinergic activation to induce the clustering of HIV co-receptors, CXCR4 and CCR5, to increase viral entry.

To determine whether HIV also induces a similar clustering to gp120, we performed similar experiments using PBMCs (infected with NL4-3) and macrophages (infected with pNL(AD_8_)). Uninfected samples did not show any co-receptor clustering as expected (Supplementary Figure 6A, control, and 6B quantification); however, inoculation with HIV_NL4-3_ induced clustering of CXCR4 (Supplementary Figure 6A and B, quantification, HIV, *p≤0.005 compared to control conditions, n=4). Similar results were observed in macrophages infected with pNL(AD_8_) (Supplementary Figure 7A and B, n=4, *p≤0.005 compared to untreated condition). In PBMCs and macrophages, blocking Panx-1 *via* pre-addition of Probenecid (Prob) or Panx-1 mimetic peptide (^10^Panx) prevented the observed induced clustering of both CXCR4 and CCR5 induced by the strains of HIV as well as the redistribution of Panx-1 in some cases especially in cultures of macrophages, probably due to the inter-individual variability (Supplementary Figures 6A and B, 7A and B, n=4, #p≤0.005 compared to HIV only condition). The pre-addition of scrambled peptide (Scr pep) did not affect gp120 or HIV-mediated clustering (Supplementary Figure 6 and Supplementary Figure 7, Scr peptide).

We must emphasize that co-receptor clustering observed in this report depended on the tropism of the gp120 used, either X4 (LAV) or R5 (BaL), and that the observed co-receptor aggregation also colocalizes with Panx-1 (Figure 5C for LAV gp120 and 5D for BaL gp120). Pearson’s colocalization index indicated that there was no CXCR4-Panx-1 colocalization in the NL4-3 inoculated PBMCs (Supplementary Figure 6A and C), but we observed strong CCR5-Panx-1 colocalization in the pNL(AD8) inoculation experiment (Supplementary Figure 7A and C, n=4). Overall, activation of Panx-1 channels by gp120 or HIV induced the clustering of CXCR4 and CCR5 in association with Panx-1 channel opening, ATP secretion, purinergic activation, and viral entry.

### Panx-1 opening induced by HIV is essential for efficient reverse transcription

One of the next critical steps post-HIV entry is reverse transcription, which could serve as a marker of successful viral entry; therefore, we evaluated the products of the enzyme that mediate this step, reverse transcriptase (RT) products, as a read-out of successful entry. A recent study demonstrated that significant early RT product synthesis occurs at 48 h post-infection, and for late RT product, it is at 72 h post-infection [39]. Therefore, here, we pre-incubated macrophages with Panx-1 blockers (Probenecid, Prob, or Panx-1 mimetic peptide, ^10^Panx) or scrambled peptide (Scr pep) as negative control, and we inoculated with pNL-AD_8_ (50 ng/mL). We extracted total DNA and then analyzed early and late RT products after 48 and 72 h *via* real-time (RT)-qPCR (Figure 6A and B, respectively).

No detection of early or late RT products was observed in the control or uninfected condition as expected (Figure 6A and B, respectively, control). HIV infection resulted in early and late RT products at 48 and 72 h post-infection (Figure 6A and B, respectively, HIV, n=3, *p≤0.005 compared to the control condition). The use of Panx-1 blockers (Probenecid, Prob, or ^10^Panx peptide, ^10^Panx) abolished the detection of early and late RT products at 48 and 72 h, indicating that Panx-1 opening is essential not only for entry but also for the generation of RT products (Figure 6A and B, respectively, n=3, *p≤0.005 compared to HIV condition). In contrast, scrambled peptide did not alter the production of early and late RT products as expected (Figure 6A and B, HIV+Scr pep, n=3, *p≤0.005 compared to the control condition). In conclusion, our data demonstrated that Panx-1 opening is essential for viral entry, which leads to the generation of RT products.

### Panx-1 channel opening is essential for viral replication

**Figure 7:**
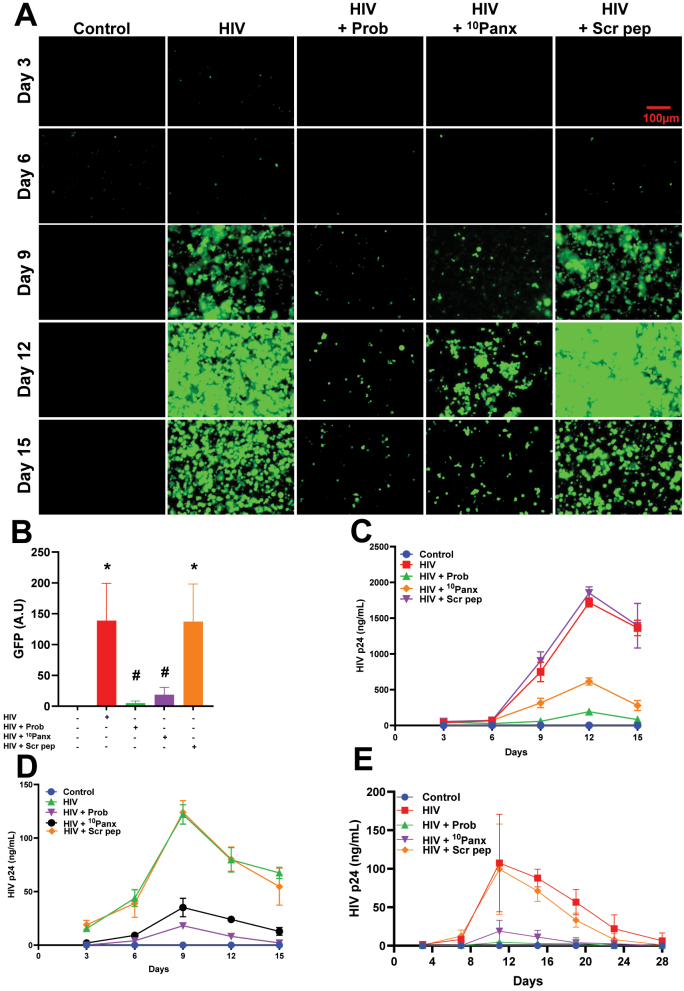
Blocking the Panx-1 channel prevents HIV replication in CEM-GFP, PBMCs, and MDMs. (A) Epifluorescence imaging of multi-day replication (15 days) of pre-treated and NL4-3 infected CEM-GFP cells, with GFP expression indicative of HIV replication. Images collected every 3 days, n=3, Scale bar=100 µm. (B) Quantification of GFP signal from all images from the experiment shown in (A) above using plate reader, n=3 and (A.U) = arbitrary unit. (C–D) p24 ELISA from pre-treated and HIV-infected CEM-GFP cells (C) and PBMCs (D) over 15 days of replication. (E) p24 ELISA from pre-treated and HIV-infected MDMs over 28 days of replication. Data are represented as mean±SD, n=3.

To examine the role of Panx-1 channel opening in viral replication, we used the cell line CEM-GFP cells, an HIV reporter cell line that, upon infection, induces GFP expression under the control of the HIV LTR (Figure 7A and B). In control uninfected conditions, no GFP expression was detected after 3, 6, 9, 12, and 15 days in culture (Figure 7A, control, representative images, and Figure 7B, GFP quantification). HIV infection with NL4-3 resulted in significant GFP production by confocal and fluorescent (Figure 7A, HIV, representative images, and Figure 7B, GFP quantification, n=3, *p≤0.005 compared to untreated condition). The addition of Probenecid (Prob) or mimetic Panx-1 peptide (^10^Panx) after entry, 12 h post-infection, to enable entry also resulted in reduced GFP production driven by reduced HIV entry and infection (Figure 7A, representative images, and Figure 7B, GFP quantification, n=4, #p≤0.005 compared to HIV condition). No changes in GFP expression induced with the virus were observed with the scrambled peptide (Figure 7A, representative images, and Figure 7B, GFP quantification, n=4, *p≤0.005 compared to the control condition). In agreement, HIV-p24 determination of the media from the same CEM-GFP cells confirms the GFP data that blocking Panx-1 channels prevents efficient replication (Figure 7C, n=4, all numbers are significant after 9 days, except control and Scrambled peptides, p≤0.005 compared to the control condition).

A similar result was found using primary PBMCs (Figure 7D, n=4, all points are significant 9–15 days except control and HIV+Prob, p≤0.005 compared to HIV condition) and macrophages (Figure 7E, n=4, all points are significant 10–18 days except control, Prob+HIV and HIV+^10^Panx, #p≤0.005 compared to HIV condition). The differences in replication are not due to changes in Panx-1 expression as examined by Western blot even after treatment with gp120 (Supplementary Figure 1). Our data indicates that Panx-1 channels are required for viral replication.

## Discussion

In this report, we demonstrate that the axis among extracellular HIV, binding to CD4/CCR5/CXCR4, the opening of Panx-1 channels, ATP secretion through the channel, and purinergic activation is required for HIV entry and replication in PBMCs, macrophages, and reporter cell lines. Panx-1 channel opening and associated purinergic signaling triggered CCR5 and CXCR4 co-receptor clustering, an essential step in viral entry and subsequent replication. Our findings fill a gap in knowledge about the role of several host components in HIV entry and replication and open the question about the identification of other channels in the pathogenesis of HIV.

Panx-1 forms a large ionic channel that is expressed by most cells and typically is in a closed conformation but opens during inflammation or pathological conditions, including ischemia-induced seizures, tumorigenesis, neuropathic pain, and HIV [48], [49], [50]. A previous report from our laboratory demonstrated that chemokines that bind to CCR5 and CXCR4 trigger a transient Panx-1 channel opening (peak between 3 and 5 min) [25]. However, gp120 and HIV have different effects; Panx-1 channel opening is extended and depends on the viral cycle (5–48 h). In both cases, membrane rearrangement was observed, but the role of membrane polarization was unknown. When HIV infects immune cells, the envelope protein gp120 binds to the host CD4 and then to the CCR5/CXCR4 receptors, depending on the viral tropism [51], [52], [53], [54], [55], [56]. In addition to this well-described mechanism, signaling in response to gp120 binding to CD4 and CCR5/CXCR4 results in increased intracellular calcium and G-protein signaling [52] as well as the opening of non-selective cation channels and calcium-activated K^+^ channels [40], 57]. Most of these channels are G protein-coupled inwardly rectifying potassium channels, a similar family to the mechanism presented by CCR5/CXCR4 for migration. For example, the ATP-sensitive potassium (K_ATP_) channels, widely distributed, are regulated by the intracellular pool of adenine nucleotides linking cellular metabolism with membrane excitability. This is important because, during the early stages of HIV infection, the p2 peptide from the Gag polyprotein increases cellular ATP levels in the cytoplasm to facilitate reverse transcription [58]. Thus, the interplay between viral infection, channels activation including Panx-1 channels, ATP, and purinergic activation are essential for HIV infection and replication.

Also, we identified in previous studies [13], 15], 31] and here that Panx-1 channels become open (Figure 1B–D), but their role in HIV infection and replication is poorly understood. It is estimated that four to six CCR5 receptors and several CD4 receptors are required to cluster to bind several HIV env proteins to form a fusion pore [59], 60]. However, the probability of several CD4 and CCR5/CXCR4 molecules aggregating at the plasma membrane is extremely low [61], [62], [63], suggesting that the process requires a mechanism involving lipid rafts and actin rearrangement. Here, we demonstrated that Panx-1 channels become open through the interaction of gp120 with CD4 and the respective chemokine receptor, CXCR4 or CCR5, resulting in ATP secretion and subsequent purinergic activation. The signaling triggered by ATP is essential for co-receptor clustering, entry, generation of RT products, and replication.

Then why is channel biology not considered in the HIV cell cycle? Plasma membrane channels are the sensors and regulators of the cell. The lack of publications in the area of channel biology in infectious diseases is due to multiple barriers related to special facilities, training, and equipment set up to protect the investigators from potential exposure to infectious reagents. In addition, most investigators in HIV are focused on genome-wide screens and protein–protein interactions; however, these methods do not detect channels or associated signaling pathways [64]. Plasma membrane channels are involved in all aspects of physiology and pathology. Therefore, we believe HIV is no different and requires more investigation. In agreement, few studies demonstrated that extracellular ATP, which can act as a “find me” signal, is released at the early stages of HIV infection and reported to be elevated in sera of PLWH [33], 34]. Extracellular ATP has been suggested to be chemotactic for some immune cells, and it activates inflammasome, which leads to the release of inflammatory cytokines like IL-1β and IL-18 *via* induced caspase-1 maturation [14], [65], [66], [67]. Further, extracellular ATP has been shown to exert autocrine and paracrine effects on cells [68]. In HIV, ATP and adenosine receptors have been associated with HIV progression because CD39 expression was associated with a delay in the onset of AIDS [69]. Also, the CD39/CD73 ratio of γδ T cells correlates with immune activation and HIV progression [70]. Similar results were identified by our collaborators, showing that CD73 is a biomarker of HIV latency [71]. Interestingly, the ATP/purinergic receptors axis in viremic individuals with a reduced CD8 T cell population has been observed [72], 73]. In B cells from viremic individuals, low CD73 and CD39 expression is associated with low CD4+ T cell counts [74]. In addition, elite controllers had CD39 and CD73 protein levels similar to uninfected individuals, and expression was compromised in viremic individuals, suggesting an important role for ATP and its processing in HIV infection and replication control [70]. In agreement, elite controllers who maintain low CD39 levels produce less IL-10 than viremic HIV-infected individuals. Also, the expression of CD39/CD73 in viremic individuals is associated with IL-10 production [70]. In agreement, the activation of purinergic receptors with pre-treatment of either BzATP or ATPγS increased HIV entry, while inhibiting purinergic receptors with oATP significantly reduced HIV entry, see Figure 4C [7], 27], 28], 75], 76]. This observation agrees with other studies that have shown the role of purinergic signaling in HIV entry and replication [7], 27], 76]. In addition to its role in HIV entry, a study has also suggested a role for extracellular ATP in HIV release from virus-containing compartments in macrophages [77], which would also mean Panx-1 could play a role in both HIV entry and release. While this is interesting, we did not specifically explore HIV budding or release in our study, but it might have contributed to decreased replication in conditions where we blocked Panx-1 in our replication experiments (Figure 7E). This will not be surprising as some of the ESCRT III complex components are ATPases that utilize ATP for the release of budding virions and the recycling of other ESCRT components [78], [79], [80], [81], [82]. However, this must be examined further, as there is elevated extracellular ATP in PLWH when compared to their uninfected peers [31]. All these data indicate that long-term ATP and ecto-ATPase dysregulation can control HIV infection and replication but also play a critical role in chronic inflammation observed in HIV-infected individuals.

Only a few manuscripts described the role of the axis between HIV and Panx-1/ATP/purinergic receptors. Previously, we have demonstrated in PBMCs that HIV binding to CD4 and/or the initial engagement with the co-receptors triggers Panx-1 channel opening and ATP secretion [13]. In Human macrophages, purinergic receptors such as P2X1, P2X7, and P2Y1 have been suggested to be essential for entry and viral replication [7]. P2X1 has been proposed to prevent binding of HIV to CCR5 and CXCR4 [76]. Extracellular ATP induces the release of viral particles from human monocyte-derived macrophages [77]. The best-known function for extracellular ATP corresponds to cell migration or polarization. Experiments in T cells show that localized ATP release in response to chemokines induces autocrine signaling by activation of P2X4 and P2Y11 receptors [83], 84]. The migratory process begins with the stimulation of the CXCR4 chemokine receptor by a Panx-1 channel-dependent mechanism [25], 85] mediated by calcium, resulting in cell polarization and receptor clustering [83]. However, the mechanism involved in these processes is poorly examined. Here, we have demonstrated that the binding of gp120 or HIV to immune cells (PBMCs, macrophages, THP-1, and CEM-GFP cell line reporter) triggers Panx-1 channel opening, sustained ATP secretion, and purinergic receptor activation, resulting in CXCR4/CCR5 clustering to enable proper HIV entry, RT activity, and replication – all essential points for HIV infection, silencing reactivation, and cure efforts.

Importantly, here, we demonstrate that both R5 and X4 gp120s and HIV induce co-receptor clustering depending on the tropism of the gp120 protein or the virus, suggesting that there must be a common activator of the observed co-receptor clustering in response to Panx-1 opening. One of the unexpected observations is in Figure 5C, when we treated the cells with X4-tropic gp120 (LAV). While we observed strong colocalization between CXCR4 and Panx-1 as expected, we also saw colocalization of CCR5-Panx-1 colocalization in this treatment as well, despite the gp120 used being an X4-tropic gp120. We think that the gp120 (LAV) triggered some response from CCR5 relative to Panx-1 in these cells, and this could be due to some unknown common activation of CCR5 and CXCR4 clustering. Interestingly, we did not observe the reverse when we treated with R5-tropic gp120 (BaL) in Figure 5D. Examination of some critical work on HIV entry and receptor usage has identified R5-tropic viruses that infect cells of myeloid origin like macrophages and microglia as mostly CD4_lo_ and X4-tropic viruses as CD4_hi_ [86]. Few studies have suggested that co-receptor clustering is important to HIV entry, and it is promoted *via* Src family tyrosine kinases, phosphatidylinositol kinases, and others [44], 87]. Also, a recent study suggested that the purinergic receptors, upon activation, interact with filamin A, one of the F-actin modulators, which reorganizes cortical actin and causes the aggregation of the co-receptors [47]. One of the observations we made in this study is the colocalization of Panx-1 and the co-receptors upon gp120 or HIV exposure. This shows that Panx-1 is an active player in this process of co-receptor clustering along with other downstream proteins like purinergic receptors because our work shows that Panx-1, not Cx43, is the main extracellular ATP release channel that is critical for entry, as demonstrated by the effect of blocking it and the release of ATP via Panx-1 is critical for co-receptor clustering. However, one question that persists is how the co-receptor that clusters is specific to the tropism of the gp120 or the HIV. More studies would have to be done to identify how the cell discriminates between the two co-receptors for clustering depending on the virus tropism. Also, the use of a mimetic peptide (^10^Panx1) without any cytotoxic effect on the cells shows that targeting Panx-1 or downstream mediators of co-receptor clustering could potentially be a tool for blocking HIV entry, which could transition to blocking infection.

Our previous data in human samples (serum and CSF) indicates that all HIV-infected individuals, even during full suppression, show signs of spontaneous Panx-1 channel opening and ATP secretion/accumulation in tissues and fluids [31]. The increased ATP levels in the circulation were highly associated with cognitive decline in the HIV-infected population, suggesting constant and chronic inflammation [31]. Normally, extracellular ATP activates P2 receptors to trigger the immune response; later, ATP is converted to adenosine by CD39, CD73, and other ectoenzymes, resulting in an anti-inflammatory response [88]. For example, CD39/CD73 expression in regulatory T cells (Tregs) converts ATP to adenosine, suppressing T effector cell function [89]. CD39 knockout mice possess enhanced immune responses due to increased ATP levels and decreased adenosine production, resulting in more CD8 T cells and resistance to bacterial infection [90]. However, in both acute and chronic HIV infection, Panx-1 is open, resulting in high circulating ATP levels, enhanced immune activation, and inflammation. Therefore, we propose that continued Panx-1 opening and elevated extracellular ATP levels will result in immune exhaustion, leading to compromise of several organs, including the brain.

We conclude that HIV induces Panx-1 channel opening, ATP secretion, and purinergic activation to participate in HIV entry and other steps of replication. Blocking any of these steps prevented HIV entry, the generation of RT products, and replication. Our data indicates that ATP secretion dysregulation is a significant contributor to HIV infection but also systemic inflammation. Our findings have clinical implications because new therapies can be added to prevent new infections and potentially prevent reactivation of viral reservoirs in line with the HIV-cure effort.

## Supplementary Material

Supplementary Material Details
