# Supplementary material for: Pannexin-1 channels, extracellular ATP, and purinergic receptors are essential for CCR5/CXCR4 clustering and HIV entry
Source: NeuroImmune Pharm Ther. 2025 May 23;4(2):217–36. doi: 10.1515/nipt-2025-0005 (PMC12304881; doi:10.1515/nipt-2025-0005)
Supplement: Supplementary file 1 — Supplementary Material Details [file j_nipt-2025-0005_suppl_001.docx]

**Supplemental Figures**

**
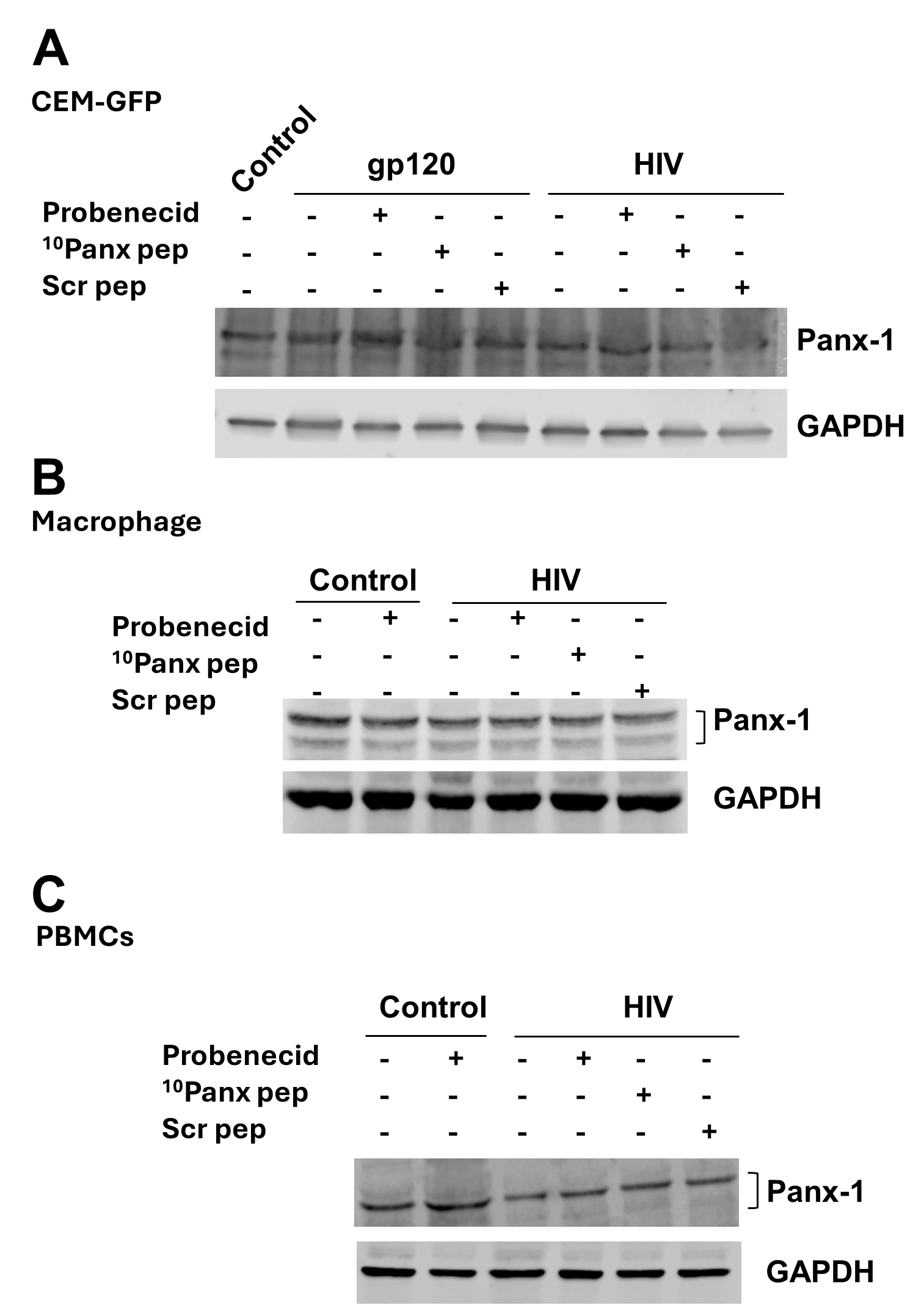
**

**Supplementary Figure 1. Immunoblot of CEM-GFP, PBMCs, and MDMs.** (A-C) Immunoblot analysis to determine levels of Panx-1 expressed relative to GAPDH in CEM-GFP cells (A), PBMCs (B), and MDMs (C) in all conditions examined with either gp120-LAV treatment (200 ng/ml) and/or HIV infected, NL4-3 for CEM-GFP and macrophages and PBMC pNL(AD_8_) in (b), n = 3.


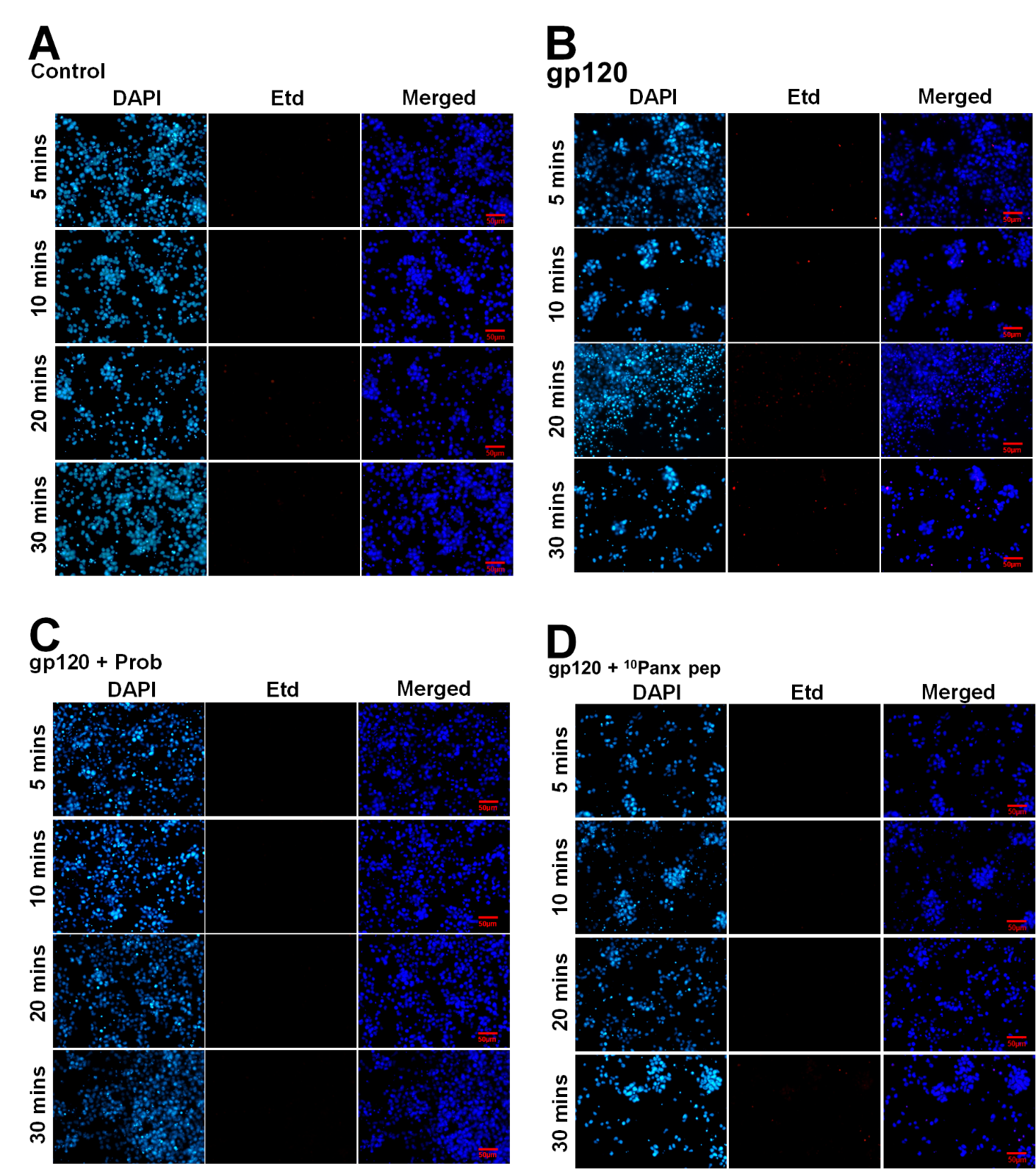


**Supplementary Figure 2. Etd uptake assay in gp120 exposed PBMCs.** (A-D) Epifluorescence imaging of pre-treated PBMCs exposed to gp120 was collected every 5 minutes for 30 minutes and quantified in (Fig 2), where all conditions are compared, n = 3. Note: Images from gp120 + Scr peptide are not shown.


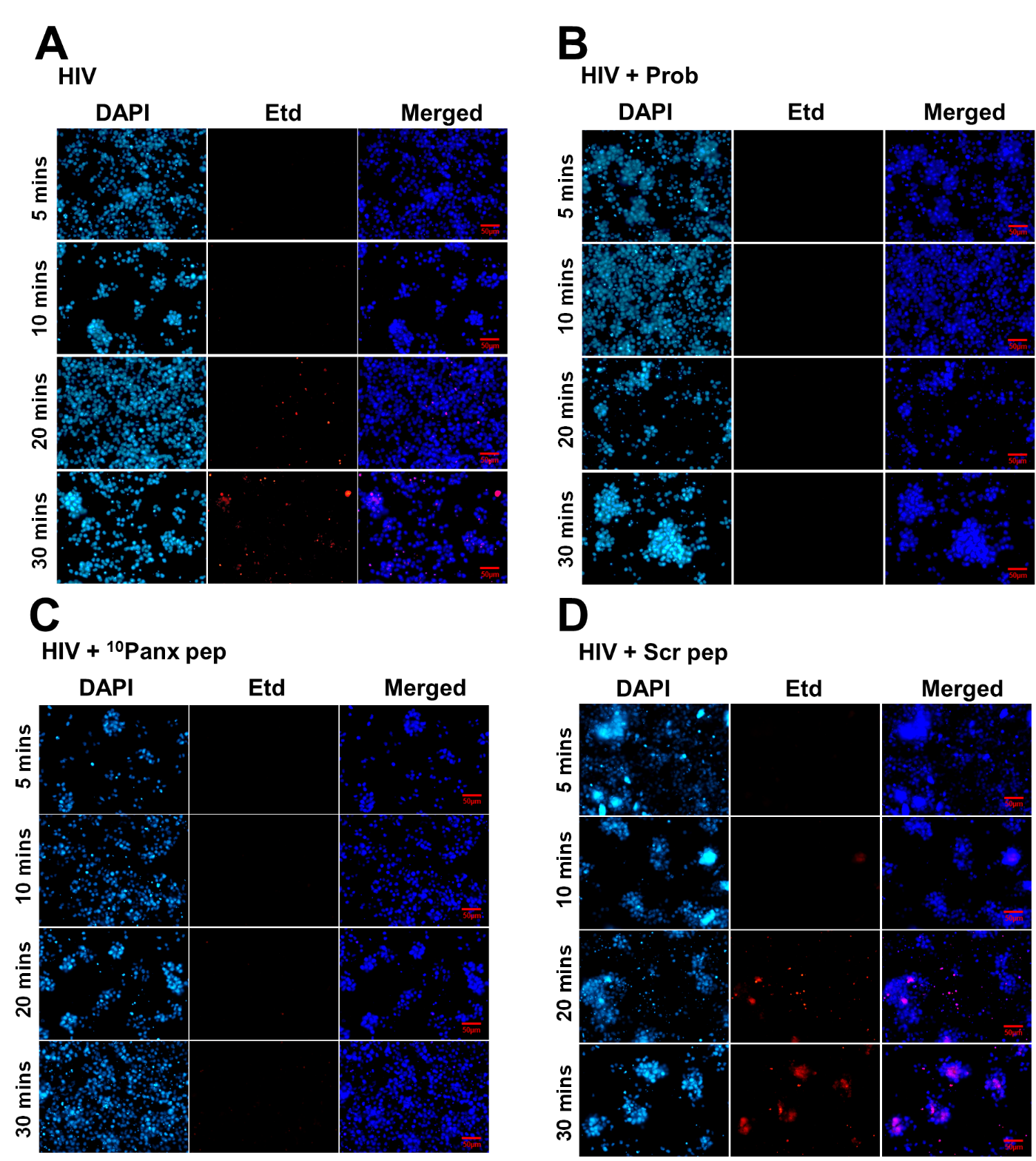


**Supplementary Figure 3. Etd uptake assay in HIV infected PBMCs.** (A-D) Epifluorescence imaging of pre-treated PBMCs infected with NL4-3 was collected every 5 minutes for 30 minutes and quantified in (Fig 2), where all conditions are compared, n = 3.


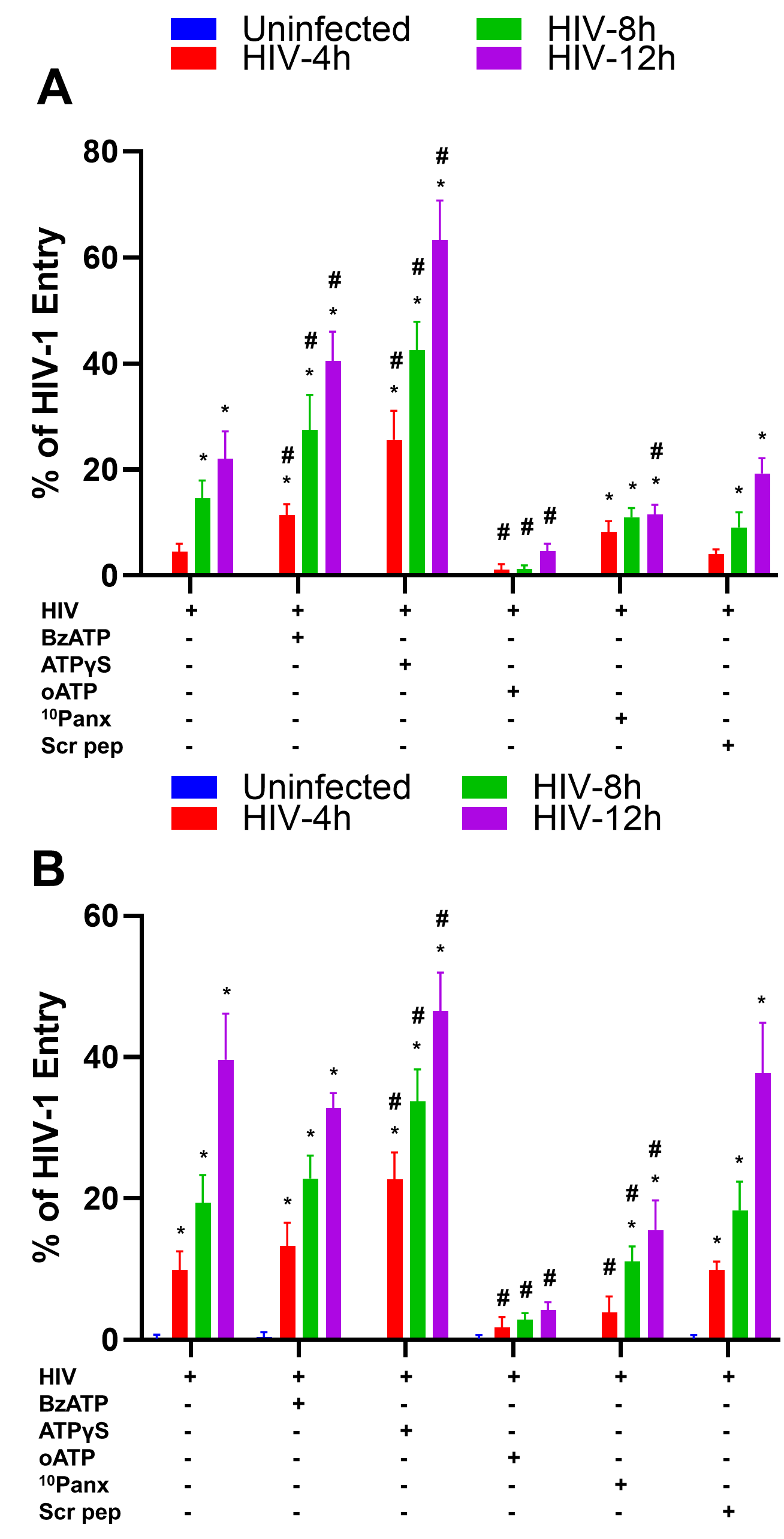


**Supplementary Figure 4. HIV-1 entry assay in MDMs from other individuals.** (A-B) MDMs from two individuals were pre-treated with BzATP (300µM), ATPγS (15µM), oATP (200µM), _10_-Panx peptide (300µM), and Scrambled (Scr) peptide (300µM), and inoculated with β-lactamase containing pseudo-typed virus (pWT/BaL-pMM310) to test for HIV entry at: 4, 8, and 12 hours post-inoculation, n = 4. % of HIV-1 Entry is determined as; number of cells positive at 460nm/number of cells positive at 528nm x 100 similar to Fig. 4C. Data are represented as mean ± S.D., * p≤ 0.005 compared to controls and # p≤ 0.005 compared to HIV.


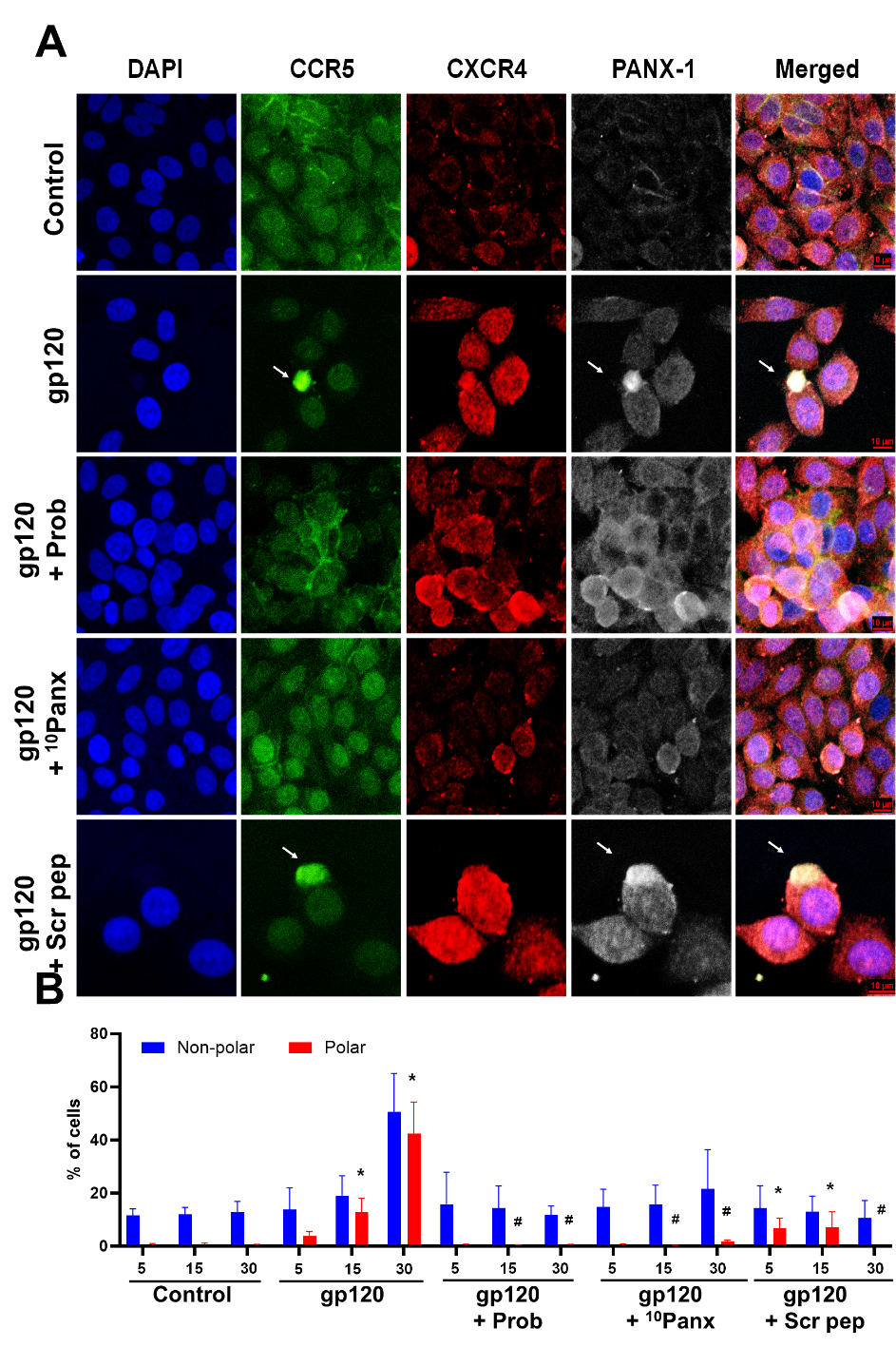


**Supplementary Figure 5. Blocking Panx-1 in MDMs prevents CCR5 clustering.** (A) Confocal images of pre-treated MDMs exposed to BaL (R5) gp120 and stained for DAPI, CCR5, CXCR4, and Panx-1. Arrow indicates the clustering of the protein of interest, and the scale bar = 10µm, n = 3. (B) Quantification of co-receptor clustering in cells displayed in (A) above determined as % of cells with or without clustering. Data are represented as mean ± S.D., * p≤ 0.005 compared to controls and # p≤ 0.005 compared to HIV.


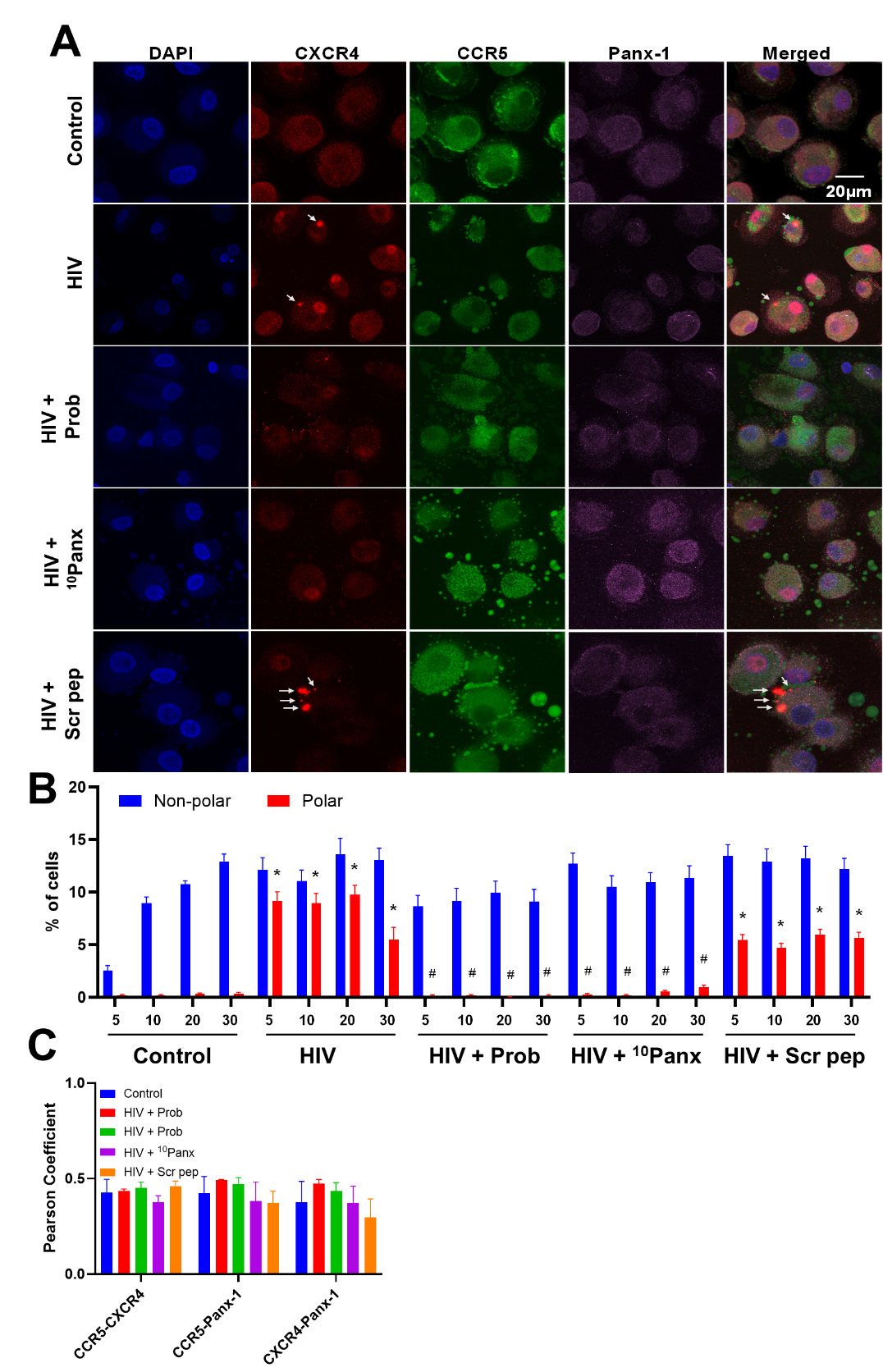


**Supplementary Figure 6. Blocking of Panx-1 in NL4-3 infected PBMCs prevents CXCR4 clustering.** (A) Confocal images of pre-treated PBMCs infected with NL4-3 and stained for DAPI, CXCR4, CCR5, and Panx-1. Arrow indicates the clustering of the protein of interest, and the scale bar = 20µm, n = 3. (B) Quantification of co-receptor clustering in cells displayed in (A) above determined as % of cells with or without clustering. Data are represented as mean ± S.D., * p≤ 0.005 compared to controls and # p≤ 0.005 compared to HIV. (C) Pearson colocalization coefficient of NL4-3 infected cells in (A) above shows no colocalization (< 0.5) in all conditions.


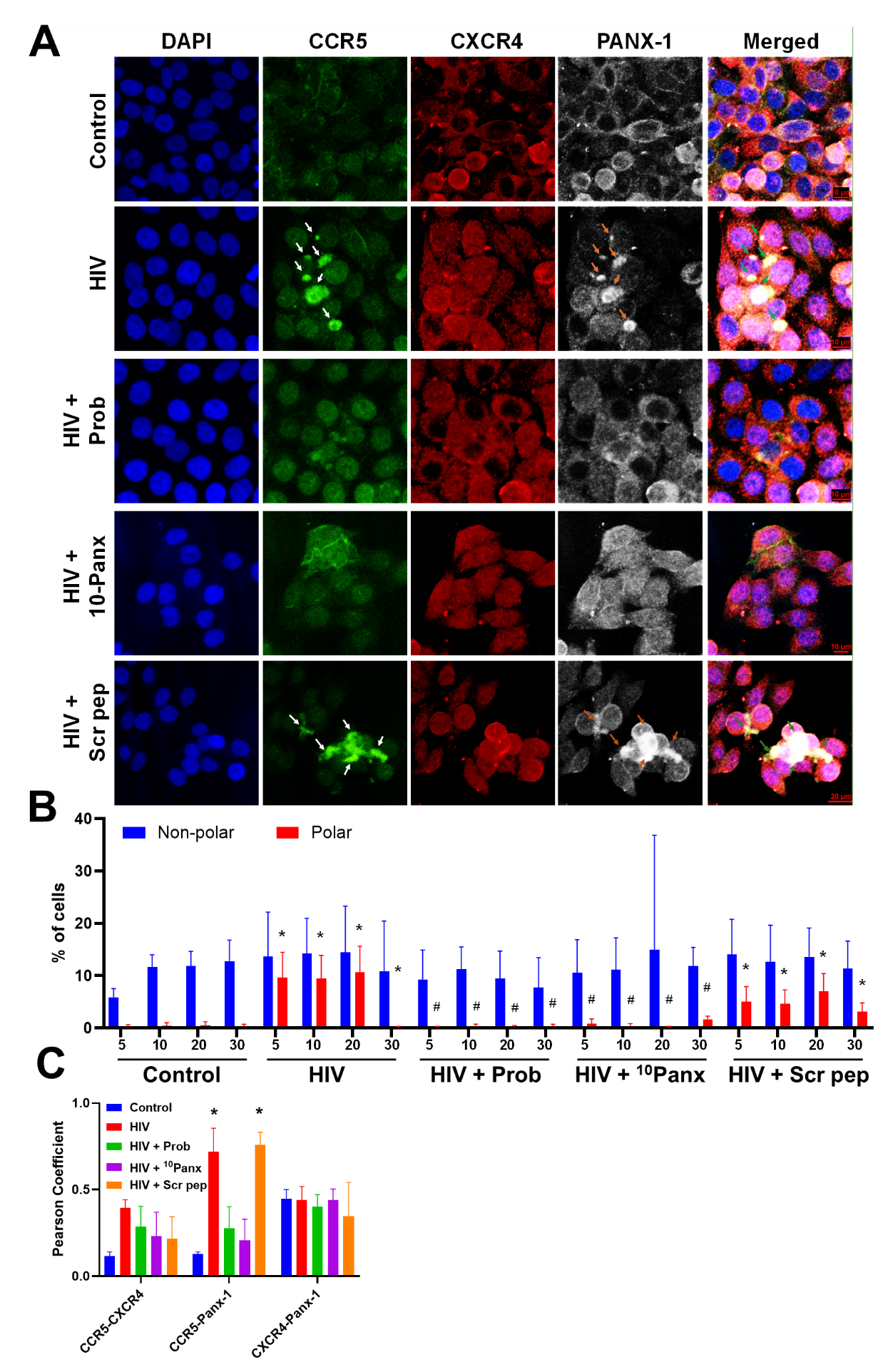


**Supplementary Figure 7. Blocking of Panx-1 in pNL(AD_8_) infected MDMs prevents CCR5 clustering.** (A) Confocal images of pre-treated MDMs infected with pNL(AD_8_) and stained for DAPI, CXCR4, CCR5, and Panx-1. Arrow indicates the clustering of the protein of interest, and the scale bar = 20µm, n = 3. The arrow indicates the clustering of proteins of interest. (B) Quantification of co-receptor clustering in cells displayed in (A) above determined as % of cells with or without clustering. Data are represented as mean ± S.D., * p≤ 0.005 compared to controls and # p≤ 0.005 compared to HIV. (C) Pearson colocalization coefficient of pNL(AD_8_) MDMs in (A) above shows CCR5-PANX-1 colocalizing (> 0.5) in HIV only and HIV + Scr peptide conditions and no colocalization in Probenecid and _10_-Panx peptide conditions.
